# Hyd/UBR5 defines a tumor suppressor pathway that links Polycomb repressive complex to regulated protein degradation in tissue growth control and tumorigenesis

**DOI:** 10.1101/gad.351856.124

**Published:** 2024-07-01

**Authors:** Pei Wen, Huiyan Lei, Hua Deng, Su Deng, Carla Rodriguez Tirado, Meiling Wang, Ping Mu, Yonggang Zheng, Duojia Pan

**Affiliations:** 1 Department of Physiology, Howard Hughes Medical Institute, University of Texas Southwestern Medical Center, Dallas, Texas 75390, USA;; 2 Department of Molecular Biology, Harold C. Simmons Comprehensive Cancer Center, University of Texas Southwestern Medical Center, Dallas, Texas 75390, USA

**Keywords:** tumor suppressor, E3 ubiquitin ligase, hyperplastic disc, UBR5, Lines, LINS1, Bowl, OSR1/2, Polycomb repressive complex, prostate cancer

## Abstract

Tumor suppressor genes play critical roles in normal tissue homeostasis, and their dysregulation underlies human diseases including cancer. Besides human genetics, model organisms such as *Drosophila* have been instrumental in discovering tumor suppressor pathways that were subsequently shown to be highly relevant in human cancer. Here we show that hyperplastic disc (Hyd), one of the first tumor suppressors isolated genetically in *Drosophila* and encoding an E3 ubiquitin ligase with hitherto unknown substrates, and Lines (Lin), best known for its role in embryonic segmentation, define an obligatory tumor suppressor protein complex (Hyd–Lin) that targets the zinc finger-containing oncoprotein Bowl for ubiquitin-mediated degradation, with Lin functioning as a substrate adaptor to recruit Bowl to Hyd for ubiquitination. Interestingly, the activity of the Hyd–Lin complex is directly inhibited by a micropeptide encoded by another zinc finger gene, *drumstick* (*drm*), which functions as a pseudosubstrate by displacing Bowl from the Hyd–Lin complex, thus stabilizing Bowl. We further identify the epigenetic regulator Polycomb repressive complex1 (PRC1) as a critical upstream regulator of the Hyd–Lin–Bowl pathway by directly repressing the transcription of the micropeptide *drm*. Consistent with these molecular studies, we show that genetic inactivation of Hyd, Lin, or PRC1 resulted in Bowl-dependent hyperplastic tissue overgrowth in vivo. We also provide evidence that the mammalian homologs of Hyd (UBR5, known to be recurrently dysregulated in various human cancers), Lin (LINS1), and Bowl (OSR1/2) constitute an analogous protein degradation pathway in human cells, and that OSR2 promotes prostate cancer tumorigenesis. Altogether, these findings define a previously unrecognized tumor suppressor pathway that links epigenetic program to regulated protein degradation in tissue growth control and tumorigenesis.

## Abstract (precis)

In this study, Wen et al. provide mechanistic insights into the regulation of the oncoprotein BOWL, which is marked for ubiquitin-mediated degradation by the E3 ligase HYD when complexed with the substrate adaptor LIN, highlighting a putative tumor-suppressive role for the HYD–LIN complex. Their findings define a previously unrecognized tumor suppressor pathway that links the epigenetic program to regulated protein degradation in tissue growth control and tumorigenesis.

An important hallmark of cancer is the ability of cancer cells to evade the powerful tumor-suppressing mechanisms in normal tissues (Hanahan and Weinberg 2011). Like many other evolutionarily conserved biological processes, model organisms such as *Drosophila* have played a critical role in uncovering fundamental mechanisms of cell proliferation and tissue growth control that are highly relevant to our understanding and treatment of human cancer (Edgar 2006; Hariharan and Bilder 2006; Andersen et al. 2013). A powerful approach to isolating tumor suppressor genes in *Drosophila* involves inducing random mutations throughout the genome and identifying those mutations that lead to tissue overgrowth in somatic mutant clones or homozygous mutant animals. These tumor suppressors then can be placed into specific signaling pathways using a combination of genetics, biochemistry, and cell biology. In the past decades, this strategy has been applied successfully to elucidate key signaling pathways, such as the Hippo pathway, that were later shown to play a conserved role in mammalian growth control and tumorigenesis (Harvey and Tapon 2007; Halder and Johnson 2011; Zheng and Pan 2019).

Despite the impressive progress in elucidating tumor suppressor pathways in *Drosophila*, some genes isolated by this approach have remained as “orphan” tumor suppressors to date, without being linked to specific signaling pathways. One such example is hyperplastic disc (Hyd). *hyd* was isolated over four decades ago in a genetic screen for homozygous mutant animals that cause imaginal disc overgrowth, representing one of the first tumor suppressor genes in *Drosophila* (Martin et al. 1977). Although it encodes a HECT family E3 ubiquitin ligase (Mansfield et al. 1994), neither the physiological substrates nor the upstream regulators of Hyd are known at present. Interestingly, UBR5, the human homolog of Hyd, was reported to be recurrently dysregulated in multiple cancer types (Shearer et al. 2015). Thus, elucidating the signaling pathway mediated by Hyd/UBR5 may offer new insights into normal tissue growth in development/homeostasis and abnormal tissue growth in tumorigenesis.

*lines* (*lin*) encodes an evolutionarily conserved protein lacking any known functional motifs (Hatini et al. 2000). It was first isolated as a segment polarity mutation affecting embryonic patterning from the landmark Nüsslein-Volhard–Wieschaus screen (Nüsslein-Volhard et al. 1984) and has since been implicated in additional cell fate specification in the embryonic midgut (Harbecke and Lengyel 1995; Johansen et al. 2003), imaginal discs (Nusinow et al. 2008; Benitez et al. 2009), and male germline hub cells (Dinardo et al. 2011). Unlike the other segment polarity genes, which encode components of the Hedgehog or Wnt signaling pathways, Lin is best known to function in a double-negative genetic hierarchy involving two Odd-skipped family zinc finger proteins, Bowl and Drumstick (Drm) (Johansen et al. 2003; Hatini et al. 2005). Bowl contains five C2H2-type zinc fingers (Wang and Coulter 1996) and has been suggested to modulate gene expression by sequestering the transcriptional corepressor Groucho (Benitez et al. 2009), whereas Drm is a 88 amino acid micropeptide containing a C2H2-type and a C2HC-type zinc finger (Green et al. 2002). Lin binds to Bowl in a manner dependent on Bowl's first zinc finger and inhibits the steady-state accumulation of Bowl protein through unknown mechanisms, whereas Drm competes with Bowl for Lin binding to promote Bowl accumulation (Hatini et al. 2005). Accordingly, in many developmental contexts studied to date, loss of Lin results in phenotypes opposite to those of the loss of Bowl or Drm, whereas overexpression of Lin phenocopies loss of Bowl or Drm (Johansen et al. 2003; Hatini et al. 2005; Nusinow et al. 2008; Benitez et al. 2009). Interestingly, mutations of *LINS1*, the human homolog of *lin*, are associated with intellectual disability from analyses of independent pedigrees in multiple ethnicities (Akawi et al. 2013; Sheth et al. 2017; Muthusamy et al. 2020; Neuhofer et al. 2020; Zhang et al. 2020a; Chen et al. 2021). At present, both the biochemical basis of the Drm–Lin–Bowl module and its functional role in tissue growth control are unknown.

In this study, we provide genetic and biochemical evidence that link Hyd and the Drm–Lin–Bowl module in a tumor suppressor pathway controlling tissue growth and tumorigenesis. We further show that the Hyd–Lin tumor suppressor pathway is regulated by Polycomb repressive complex1 (PRC1) through epigenetic silencing of Drm expression and is functionally required for PRC1's tumor suppressor activity in vivo. We also provide evidence that the mammalian homologs of Hyd (UBR5), Lin (LINS1), and Bowl (OSR1/2) constitute an analogous protein degradation pathway in human cells, and that OSR2 promotes prostate cancer tumorigenesis. Our study thus elucidates a new tumor suppressor pathway that couples an epigenetic program to tissue growth and tumorigenesis with implications in human cancer.

## Results

### Identification of *lin* as a tumor suppressor gene in *Drosophila*

**Figure 1.**
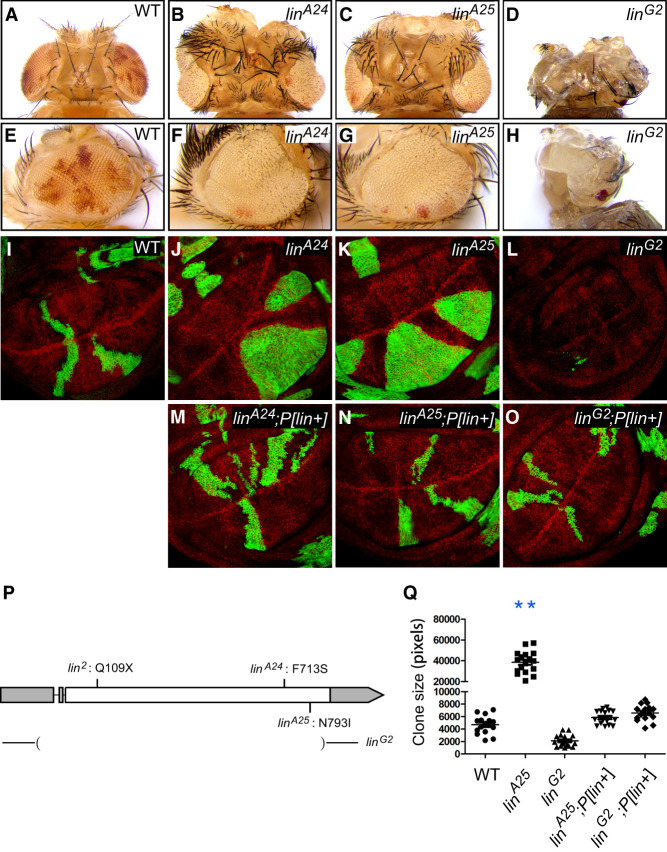
Identification of *lin* as a tumor suppressor gene in *Drosophila*. (*A–H*) Adult eyes composed of tissues predominantly of the indicated genotype, generated by the ey-FLP/recessive cell-lethal technique. Mutant tissues are marked by the lack of red pigment. (*A–D*) Top view. (*E–H*) Side view. Unlike *lin*^*A24*^ and *lin*^*A25*^, only pharate adults could be recovered for *lin*^*G2*^. Note the enlarged head and eye size of *lin*^*A24*^ and *lin*^*A25*^, in contrast to the diminished head and eye size of *lin*^*G2*^. Also note the increased representation of white tissues in *lin*^*A24*^ and *lin*^*A25*^ eyes, and the absence of white tissues in the diminutive *lin*^*G2*^ eyes. (*I–L*) Third instar wing discs containing GFP-positive mutant clones of the indicated genotype, generated by *hs*-FLP induction of MARCM (mosaic analysis with a repressible cell marker) clones at 48 h after egg laying. The clones are marked by GFP expression (green). Note the increased size and round shape of *lin*^*A24*^ and *lin*^*A25*^ mutant clones. Only rare scattered *lin*^*G2*^ mutant cells survived to third instar stage. Quantification of clone size is shown in *Q*. (*M–O*) Third instar wing discs containing GFP-positive MARCM clones of different *lin* mutant alleles. The clones were generated in flies carrying a *P[lin*^+^*]* genomic rescue transgene. Note the rescue of both clone size and clone shape by the *P[lin^+^]* transgene (cf. *I–L*). (*P*) Schematic view of *lin* gene structure showing molecular lesions of various *lin* mutant alleles. Exons and introns are indicated by boxes and lines, respectively. Gray boxes indicate 5′ and 3′ UTRs, and white boxes indicate coding region. Sequencing analysis revealed F713S mutation in *lin*^*A24*^, N793I mutation in *lin*^*A25*^, Q109STOP mutation in *lin*^*2*^, and a large deletion of the coding region in *lin*^*G2*^. (*Q*) Quantification of clone size in the experiments described in *I*, *K*, *L*, *N*, and *O* (mean ± SEM; *n* ≥ 15). (**) *P* < 0.01.

In a chemical mutagenesis screen for negative growth regulators using the *eyeless*-FLP/recessive cell-lethal technique (Newsome et al. 2000), we identified two lethal mutations (*A24* and *A25*) defining a single complementation group on chromosome 2R that caused overgrowth (Fig. 1A–C) and increased the representation of mutant tissues in adult mosaic eyes (Fig. 1E–G). Consistent with the overgrowth phenotype in adult tissues, *A24* or *A25* mutant clones in third instar wing imaginal discs were also larger than the control clones (Fig. 1I–K). Furthermore, unlike control cells that intermingled with their surrounding neighbors to form irregular clonal borders, *A24* or *A25* mutant clones adopted a round shape with a smooth clonal boundary (Fig. 1I–K), a common characteristic of many overgrowth mutants in *Drosophila*.

By deficiency mapping, *A24* and *A25* failed to complement the lethality of Df(2R)ED1770, placing the mutations between molecular coordinates 8,655,629 and 9,207,541 (Supplemental Fig. S1A). We further narrowed the mutations to molecular coordinates 8,898,753; 8,922,730 based on their complementation with three deficiencies within this region: Df(2R)Exel7098 (8,649,482; 8,733,630), Df(2R)BSC269 (8,722,439; 8,898,753), and Df(2R)ED1791 (8,922,730; 9,553,252) (Supplemental Fig. S1A). Crosses with available mutations in this genomic interval showed that *A24* and *A25* failed to complement two independent alleles of *lin* (*lin*^*2*^ and *lin*^*G2*^), both considered as genetic-null or strong alleles (Hatini et al. 2000). Sequencing analysis revealed a F713S missense mutation in *A24* and a N793I missense mutation in *A25*. Both mutations result in changes of evolutionarily conserved residues in the C-terminal region of the Lin protein (Fig. 1P; Supplemental Fig. S1B), suggesting that *A24* and *A25* represent mutant alleles of *lin*. We also sequenced the two available alleles of *lin*: *lin*^*2*^ introduced a stop codon at Q109, and *lin*^*G2*^ caused a large deletion of the coding region (Fig. 1P), consistent with them being strong/null alleles.

Our isolation of *lin* mutant alleles that caused overgrowth of imaginal tissues with normal patterning was unexpected because it was reported that *lin* is required for the proper differentiation of imaginal disc epithelia. Accordingly, in mosaic clones induced in early larval development, *lin* mutant cells undergo apoptosis and rarely survive to adulthood (Nusinow et al. 2008; Benitez et al. 2009). Indeed, unlike mosaic eyes with *lin*^*A24*^ or *lin*^*A25*^, mosaic eyes containing the null mutant *lin*^*G2*^ clones were greatly reduced in size and contained only heterozygous cells (Fig. 1D,H). Likewise, when mutant clones were induced in wing imaginal discs by *hs*-FLP during the first instar larval stage, only scattered *lin*^*G2*^ mutant cells survived to the third instar stage (Fig. 1L). Another strong allele, *lin*^*2*^, showed a similar cell loss phenotype (data not shown). Despite their contrasting phenotypes, both the overgrowth of *lin*^*A24*^ and *lin*^*A25*^ clones and the poor survival of *lin*^*G2*^ clones were fully rescued by a genomic transgene covering the *lin* locus (Fig. 1M–O,Q), consistent with all these mutants being loss-of-function alleles. Taken together, the isolation of hypomorphic *lin* alleles allowed us to uncover a hidden tumor suppressor function of *lin* that would otherwise be masked by the pleiotropic effects of strong/null alleles on cell differentiation and survival. Given the similar overgrowth phenotype resulting from *lin*^*A24*^ and *lin*^*A25*^, we focused our follow-up analysis on *lin*^*A25*^.

### Lin suppresses tissue growth by inhibiting Bowl protein level and expression of the Notch pathway ligand Dl

**Figure 2.**
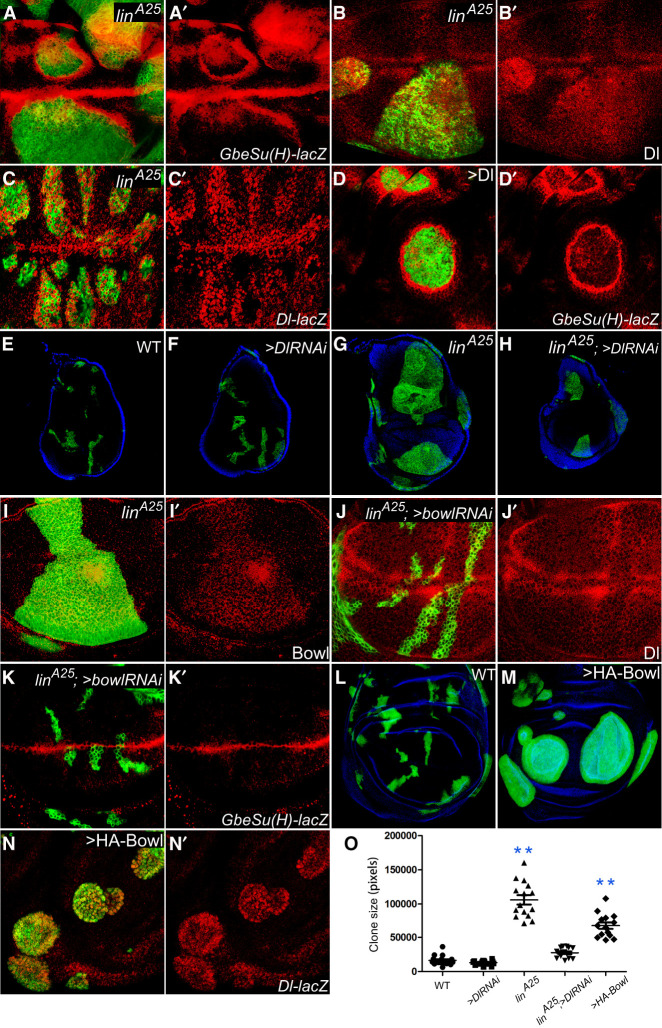
Notch ligand Dl and zinc finger protein Bowl are elevated in *lin* mutant cells and required for tissue overgrowth caused by the loss of *lin*. (*A*,*A*′) A third instar wing imaginal disc containing GFP-positive MARCM clones of *lin*^*A25*^ was stained for the Notch pathway reporter *GbeSu(H)-lacZ* (red). *GbeSu(H)-lacZ* was induced inside the mutant clones and in a stripe of cells immediately outside the clone boundary. (*B*,*B*′) A third instar wing imaginal disc containing GFP-positive MARCM clones of *lin*^*A25*^ was stained for Dl protein (red). Note the ectopically increased Dl protein level in *lin*^*A25*^ mutant clones marked by GFP. (*C*,*C*′) Similar to *B* and *B*′ except that a *Dl-lacZ* reporter (red) was examined. Note the ectopically increased *Dl-lacZ* level in *lin*^*A25*^ mutant clones. (*D*,*D*′) A third instar wing imaginal disc containing GFP-positive Flp-out clones with Dl overexpression was stained for *GbeSu(H)-lacZ* (red). Note the round shape of Dl-overexpressing clones and the induction of *GbeSu(H)-lacZ* inside the mutant clones and in a stripe of cells immediately outside the clone boundary. (*E–H*) Third instar wing discs containing GFP-positive MARCM clones of the indicated genotype, counterstained with DAPI (blue). Note the similar size of *Dl* RNAi clones compared with the wild-type control clones (cf. *E* and *F*). Also note that the overgrowth of *lin*^*A25*^ mutant clones was decreased by *Dl* RNAi (cf. *G* and *H*). (*I*,*I*′) A third instar wing disc containing GFP-positive MARCM clones of *lin*^*A25*^ was stained for Bowl protein (red). Note the modest increase of Bowl protein level in *lin*^*A25*^ clones. (*J–K*′) A third instar wing disc containing GFP-positive MARCM clones of *lin*^*A25*^ with Bowl RNAi knockdown was stained for Dl protein (red, *J,J*′) or the Notch pathway reporter *GbeSu(H)-lacZ* (red, *K*,*K*′). Note the normalization of Dl protein level, *GbeSu(H)-lacZ* reporter, clone size, and clone shape by Bowl RNAi, in contrast to *lin*^*A25*^ clones without Bowl RNAi (cf, *A–B*′). (*L*,*M*) A third instar wing disc containing GFP-positive MARCM clones without (*L*) or with (*M*) Bowl overexpression, counterstained with DAPI (blue). Note the increased size and round shape of Bowl-overexpressing clones. (*N*,*N*′) A third instar wing imaginal disc containing GFP-positive Flp-out clones with Bowl overexpression was stained for the *Dl-lacZ* reporter (red). Note the cell-autonomous induction of the *Dl-lacZ* reporter within the clones. (*O*) Quantification of clone size for the experiments described in *E–H* and *M* (mean ± SEM; *n* ≥ 15). (**) *P* < 0.01.

To pinpoint the downstream effectors underlying the novel tumor suppressor function of Lin, we systematically probed reporters for various developmental signaling pathways in *lin*^*A25*^ mutant clones in wing imaginal discs. Although we observed no obvious changes in Wnt, BMP, JAK/STAT, JNK, or Hippo signaling (Supplemental Fig. S2), the Notch pathway reporter *GbeSu(H)-lacZ* was upregulated both inside the mutant clones and, most prominently, in a stripe of cells immediately outside the mutant clones (Fig. 2A,A′). Such a pattern of Notch reporter activation suggests that the expression of Notch ligands may be increased inside the mutant clones. Consistent with this notion, *lin*^*A25*^ mutant clones showed a cell-autonomous upregulation of the Notch ligand Delta (Dl) within clone boundaries, as indicated by either Dl protein staining (Fig. 2B,B′) or a *Dl-lacZ* transcription reporter (Fig. 2C,C′). Indeed, clonal overexpression of Dl resulted in round-shaped clones, as well as the upregulation of *GbeSu(H)-lacZ* both inside the clone and in a stripe of cells immediately outside the clone boundary (Fig. 2D,D′), mimicking those observed for the *lin*^*A25*^ mutant clones. Furthermore, RNAi knockdown of Dl (using multiple UAS-RNAi lines) suppressed the overgrowth of *lin*^*A25*^ mutant clones (Fig. 2E–H, quantified in O). These results implicate Dl as a critical downstream target of Lin in tissue growth control.

Next, we asked whether the tumor suppressor function of Lin can be attributed to its regulation of Bowl activity. Consistent with this notion, we observed an increased Bowl protein level in *lin*^*A25*^ mutant clones (Fig. 2I,I′). Importantly, RNAi knockdown of Bowl (using multiple UAS-RNAi lines) not only completely suppressed the round shape and overgrowth of *lin*^*A25*^ mutant clones but also suppressed the elevated expression of Dl and the Notch reporter *GbeSu(H)-lacZ* (Fig. 2J–K′). Indeed, overexpression of Bowl was sufficient to induce clonal overgrowth (Fig. 2L,M) and cell-autonomous upregulation of Dl expression (Fig. 2N,N′), resembling those observed in *lin*^*A25*^ mutant clones. Together, these findings suggest that Lin suppresses tissue growth by inhibiting Bowl protein level and expression of the Notch pathway ligand Dl.

### Lin promotes Bowl ubiquitination and degradation

**Figure 3.**
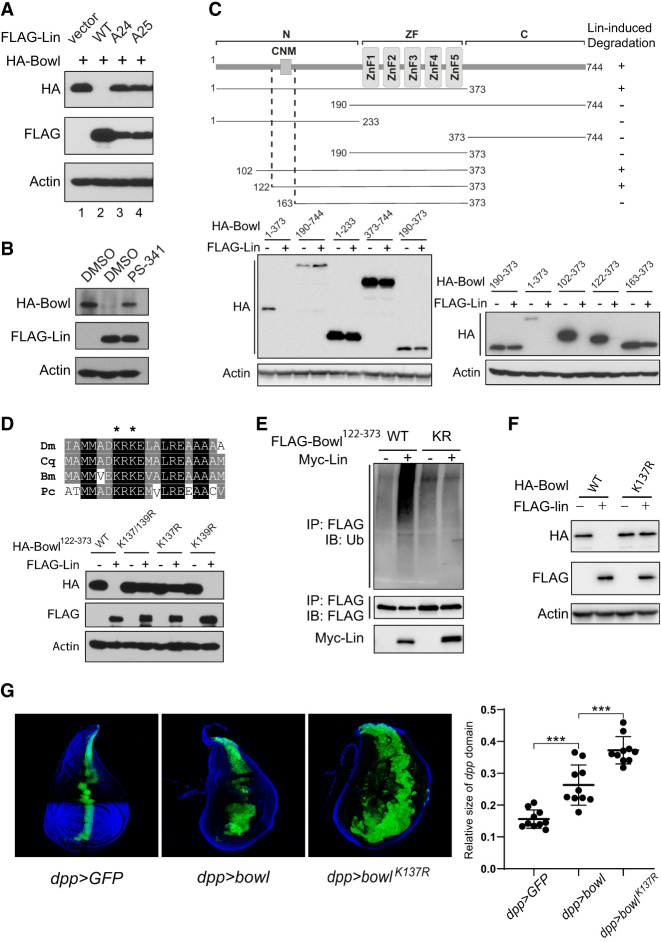
Lin destabilizes Bowl protein through the ubiquitin–proteasome pathway. (*A*) S2R^+^ cell lysates coexpressing HA-Bowl with different FLAG-Lin constructs were probed with the indicated antibodies. Note the dramatic reduction of HA-Bowl when coexpressed with Lin but not when coexpressed with Lin^A24^ or Lin^A25^ mutant protein. (*B*) S2R^+^ cells coexpressing HA-Bowl and FLAG-Lin were treated with 10 μM PS-341 or vehicle solvent (DMSO) for 4 h before analysis by Western blotting. Note the restoration of HA-Bowl by PS-341 treatment. (*C*) Dissection of Bowl regions required for Lin-mediated degradation. (*Top*) Schematic summary of Bowl deletion constructs tested for Lin-induced degradation. (*Bottom*) Western blots of S2R^+^ cell lysates coexpressing FLAG-Lin with various HA-Bowl constructs. The *left* gel shows that only a Bowl fragment containing both the N terminus and zinc finger (ZF; Bowl^1–373^) was destabilized by Lin. The *right* gel shows analysis of N-terminal truncations of Bowl^1–373^. Lin promoted the degradation of Bowl^1–373^, Bowl^102–373^, and Bowl^122–373^ but not Bowl^163–373^ or Bowl^190–373^. (*D*) Identification of Bowl^K137^ as a critical site for Lin-mediated degradation. (*Top*) Alignment of the conserved N-terminal motif (CNM) in the Bowl orthologs for different species. (Dm) *Drosophila melanogaster*, (Cq) *Culex quinquefasciatus*, (Bm) *Bombyx mori*, (Pc) *Pediculus humanus corporis*. (*Bottom*) Analysis of Lin-mediated degradation of Bowl^122–373^ constructs with mutations of the conserved lysine residues in the CNM. K137R, but not K139R, abolished Lin-mediated degradation in S2R^+^ cells. (*E*) Bowl^K137^ is required for Lin-mediated ubiquitination. S2R^+^ cells expressing FLAG-Bowl^122–373^ (or the corresponding K137R mutant) with or without Myc-Lin were treated with the proteasome inhibitor PS-341 and then immunoprecipitated with FLAG antibody. The immunoprecipitation (IP) product was probed with antiubiquitin antibody to detect Bowl ubiquitination. Lin induced ubiquitination of Bowl, but not the K137R mutant. (*F*) A full-length Bowl construct carrying the K137R mutation (Bowl^K137R^) abolished Lin-mediated degradation in S2R^+^ cells. (*G*) Third instar wing discs expressing *UAS-Bowl* or *UAS-Bowl*^*K137R*^ by the *dpp-Gal4 UAS-GFP* driver. Note the expansion of the *dpp* domain by Bowl expression, and even greater expansion by Bowl^K137R^ expression. Quantification of the size of the *dpp* domain relative to the wing size is shown at the *right* (mean ± SEM; *n* = 10). (***) *P* < 0.001.

To understand how Lin inhibits Bowl protein level, we established a Lin-dependent Bowl destabilization assay in *Drosophila* cells. When HA-tagged Bowl protein was expressed alone in S2R^+^ cells, it was easily detected by Western blotting of the cell lysate (Fig. 3A, lane 1). However, when HA-Bowl was coexpressed with Lin, the HA-Bowl protein was hardly detectable, presumably due to the destabilizing activity of Lin (Fig. 3A, lane 2). In contrast, this destabilizing effect was not observed when HA-Bowl was coexpressed with mutant forms of Lin carrying the *lin*^*A24*^ or *lin*^*A25*^ missense mutation (Fig. 3A, lanes 3,4), consistent with their loss-of-function nature. Bowl degradation was largely suppressed by treatment with PS-341, a highly specific proteasome inhibitor (Fig. 3B), suggesting that Lin destabilizes Bowl through the ubiquitin–26S proteasome pathway.

Next, we mapped the protein domain(s) in Bowl that are required for Lin-mediated degradation. Bowl contains a central region composed of five zinc fingers (ZFs) flanked by N-terminal and C-terminal regions lacking obvious functional motifs (Fig. 3C). We found that although only the ZFs, but not the N-terminal or C-terminal region of Bowl, interact with Lin (Supplemental Fig. S3A; Hatini et al. 2005), this interaction alone is insufficient for Bowl degradation (Fig. 3C). Rather, both the N-terminal region and ZFs are required for Lin-mediated degradation (Fig. 3C).

To map the N-terminal degron required for Bowl degradation, we examined a series of Bowl constructs containing ZFs and different N-terminal truncations. Although a Bowl construct carrying an N-terminal deletion to amino acid 122 (Bowl^122–373^) was still destabilized by Lin, an N-terminal deletion to amino acid 163 (Bowl^163–373^) abolished destabilization, pinpointing the region between amino acid 123 and amino acid 163 as a potential degron for Lin-mediated degradation (Fig. 3C). We noted that this region contains a sequence motif, referred to here as the conserved N-terminal motif (CNM), that is highly conserved among Bowl orthologs from multiple insect species (Fig. 3D). Of note, this CNM is not present in Drm, a distinct zinc finger protein that is known to interact with Lin but is not destabilized by Lin (Green et al. 2002). Strikingly, although Drm is normally not destabilized by Lin, attaching the CNM to Drm was sufficient to confer Lin-induced degradation to the chimeric protein (Supplemental Fig. S3B). Taken together, these results suggest that Lin-mediated Bowl degradation requires not only the ZF region that interacts with Lin but also an N-terminal motif that may function as a degron.

Interestingly, the CNM contains two highly conserved lysine residues (K137 and K139) (Fig. 3D), raising the possibility that they may function as ubiquitination sites in Lin-induced Bowl degradation. We tested this hypothesis by introducing a double or single K-to-R mutation of these lysine residues into the minimal Lin-responsive construct Bowl^122–373^. As shown in Figure 3D, both the K137/139R double mutant and the K137R single mutant greatly diminished Lin-induced degradation of Bowl^122–373^ in S2R^+^ cells, whereas the K139R mutant had no effect (Fig. 3D). Interestingly, the K137R mutant abolished not only Lin-induced Bowl^122–373^ degradation but also Lin-induced Bowl^122–373^ ubiquitination (Fig. 3E), suggesting that Lin destabilizes Bowl by promoting K137 ubiquitination and proteasome-mediated degradation.

To further test the importance of K137 in Lin-induced degradation, we introduced the K137R mutation into the full-length Bowl protein. As expected, the K137R mutation abolished Lin-induced Bowl degradation in S2R^+^ cells (Fig. 3F). Given that the Bowl^K137R^ mutant abolished Lin-induced degradation, one might expect it to behave as a gain-of-function mutant compared with wild-type Bowl. We tested this prediction by comparing the in vivo activity of UAS-Bowl and UAS-Bowl^K137R^ transgenes inserted into identical landing sites by φC31-mediated chromosomal integration (Bischof et al. 2007) using the *dpp-Gal4* driver. Indeed, compared with wild-type Bowl, expression of Bowl^K137R^ resulted in a higher protein level (Supplemental Fig. S3C) and a greater expansion of the *dpp*-expressing domain in the wing imaginal discs (Fig. 3G), further supporting the importance of Bowl^K137^ in Lin-mediated degradation.

### Identification of Hyd as the critical E3 ubiquitin ligase that mediates Lin-induced Bowl degradation

**Figure 4.**
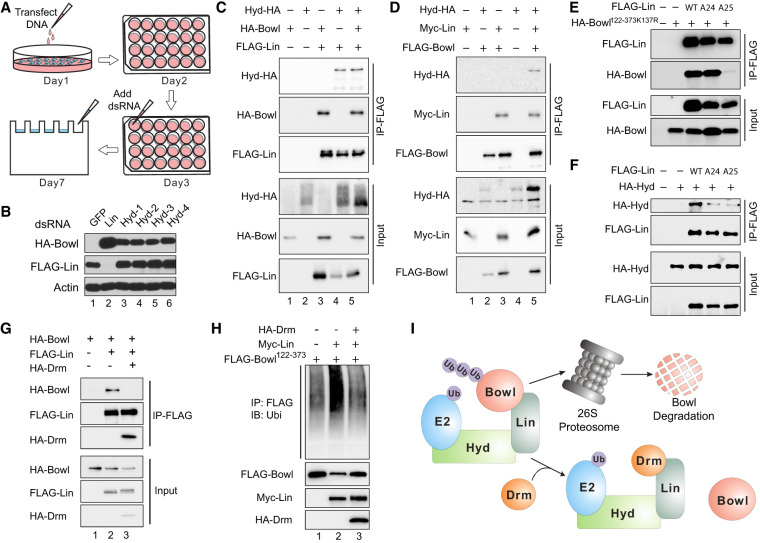
Identification of Hyd as a critical E3 ubiquitin ligase mediating Lin-induced Bowl degradation. (*A*) Schematic diagram of a pooled RNAi screen for E3 ligases required for Lin-induced Bowl degradation. S2R^+^ cells transfected with HA-Bowl and FLAG-Lin were split into 24 well plates, each well treated with a pool of dsRNAs targeting four different E3 ligases, followed by Western blot analysis of cell lysates. (*B*) Confirmation of Hyd as an E3 ligase required for Lin-induced Bowl degradation. S2R^+^ cells expressing HA-Bowl and FLAG-Lin were treated with different Hyd dsRNAs. (Hyd-1) dsRNA in the original RNAi library (Hyd-2, Hyd-3, and Hyd-4) three synthesized dsRNAs targeting different regions of Hyd. HA-Bowl was normally undetectable under such conditions due to Lin-induced degradation. However, it was stabilized by RNAi against Hyd or Lin. GFP dsRNA was included as a negative control. (*C*) S2R^+^ cells expressing the indicated constructs were subjected to coimmunoprecipitation (co-IP) assay as indicated. Cells were treated with 10 μM PS-341 for 4 h before harvesting. Interaction was readily detected between FLAG-Lin and HA-Bowl (lane *3*) and between FLAG-Lin and Hyd-HA (lane *4*). (Lane *5*) Neither pairwise interaction was affected by coexpression of the third protein. (*D*) S2R^+^ cells expressing the indicated constructs were subjected to co-IP assay as indicated. Cells were treated with PS-341 as in *C*. Co-IP between FLAG-Bowl and Hyd-HA was detected only in the presence of Myc-Lin (cf. lanes *2* and *5*). (*E*) S2R^+^ cells expressing the indicated constructs were subjected to co-IP assay as indicated. (HA-Bowl^122–373K137R^) The K137R mutant form of Bowl fragment 122–373 as described in Figure 3C. Note that the Lin–Bowl co-IP was severely impaired by *lin*^*A25*^, but not *lin*^*A24*^, mutation. (*F*) S2R^+^ cells expressing the indicated constructs were subjected to co-IP assay as indicated. Note that the Lin–Hyd co-IP was impaired by both *lin*^*A24*^ and *lin*^*A25*^ mutations. (*G*) S2R^+^ cells expressing the indicated constructs were subjected to co-IP assay as indicated. Cells were treated with PS-341 as in *C*. Note the disruption of the Lin–Bowl interaction by Drm (cf. lanes *2* and *3*). (*H*) S2R^+^ cells expressing the indicated constructs were treated with PS-341 before IP with FLAG antibody. The IP product was probed with antiubiquitin antibody to detect Bowl ubiquitination. Lin-induced Bowl ubiquitination was suppressed by Drm. (*I*) Schematic model for the regulation of Bowl degradation. In the absence of Drm, Lin promotes Bowl degradation by functioning as a substrate adaptor protein recruiting Bowl to Hyd. Drm competes with Bowl for Lin binding, thus releasing Bowl from Lin–Hyd, resulting in Bowl stabilization.

Next, we investigated the biochemical mechanism by which Lin promotes Bowl ubiquitination and degradation. Because Lin does not encode a protein with known functional motifs related to the ubiquitin–proteasome pathway, we hypothesized that Lin may function as a novel substrate adaptor protein that recruits an unknown E3 ubiquitin ligase to ubiquitinate the Bowl protein. If so, such an E3 ligase should be required for Lin-induced Bowl degradation. We therefore conducted an RNAi screen for E3 ligases that were required for Lin-dependent Bowl degradation in S2R^+^ cells (Fig. 4A). By screening a library of dsRNAs targeting all E3 ligases encoded in the *Drosophila* genome (a total of 179) (Supplemental Table S1), we identified hyperplastic discs (Hyd), which encodes a HECT domain E3 ubiquitin ligase (Mansfield et al. 1994), as the only positive hit whose RNAi abolished Lin-induced Bowl degradation (Supplemental Fig. S4A,B). This was further confirmed by three additional dsRNAs targeting different regions of Hyd (Fig. 4B). Taken together, these results implicate Hyd as a critical E3 ubiquitin ligase that mediates Lin-dependent degradation of Bowl.

To understand how Lin and Hyd function together to promote Bowl degradation, we probed physical interactions among the three proteins by coimmunoprecipitation (co-IP) in S2R^+^ cells. In pairwise co-IP assays, we observed Lin–Hyd association and Lin–Bowl association (Fig. 4C), but not Hyd–Bowl association (Fig. 4D). Interestingly, when the three proteins were coexpressed, we could readily detect Hyd–Bowl association under the same IP conditions (Fig. 4D). These findings support our hypothesis that Lin functions as a substrate adaptor protein that bridges the E3 ligase Hyd and its substrate, Bowl, to facilitate Bowl ubiquitination and degradation.

To gain additional insights into how Lin functions as a substrate adaptor protein that bridges Hyd and Bowl, we took advantage of a putative 3D structure of Lin predicted by AlphaFold (Varadi et al. 2024), which reveals a core consisting predominantly of α helices (Supplemental Fig. S4C). Interestingly, the two loss-of-function missense mutations identified in our genetic screen (*lin*^*A24*^ and *lin*^*A25*^) are located on opposite sides of this α-helical core. Consistent with their spatial positions, these two mutations differentially affect Lin–Hyd and Lin–Bow interactions. Although both mutations impaired Lin–Hyd interactions, only the *lin*^*A25*^ mutation impaired Lin–Bowl interactions (Fig. 4E,F).

We also explored how the small zinc finger protein Drm antagonizes Lin function. Coexpression of Drm inhibited not only the physical association between Lin and Bowl (Fig. 4G) but also Lin-induced Bowl degradation (Supplemental Fig. S4D) and ubiquitination (Fig. 4H). These results suggest that Drm functions as a pseudosubstrate that stabilizes Bowl by competing Bowl off the Lin–Hyd E3 ligase complex (Fig. 4I).

### *bowl* is genetically epistatic to *hyd* and *lin*

**Figure 5.**
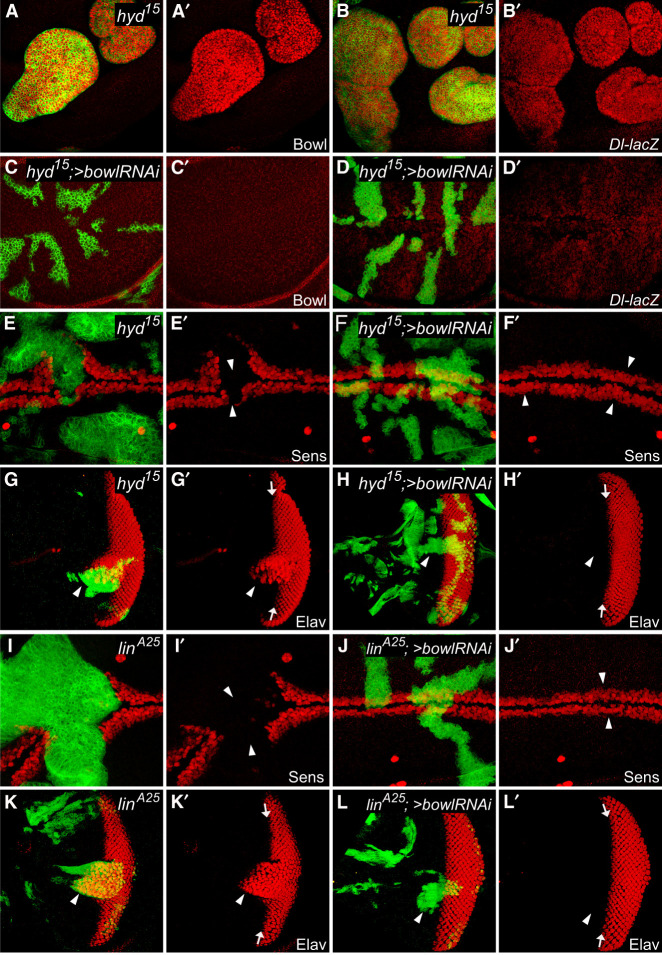
Hyd is required for Bowl degradation in vivo. (*A–B*′) A third instar wing disc containing GFP-positive MARCM clones of *hyd*^*15*^, stained for Bowl (red; *A*,*A*′) or *Dl-lacZ* reporter (red; *B*,*B*′). Note the cell-autonomous increase of Bowl protein and *Dl-lacZ* levels, and the round shape of the mutant clones. (*C–D*′) A third instar wing disc containing GFP-positive MARCM clones of *hyd*^*15*^ with Bowl RNAi was stained for Bowl (red, *C*,*C*′) or *Dl-lacZ* reporter (red, *D*,*D*′). Note the normalization of clone shape/size and *Dl-lacZ* expression upon Bowl RNAi. (*E–F*′) A third instar wing disc containing GFP-positive MARCM clones of *hyd*^*15*^ (*E*,*E*′) or *hyd*^*15*^ with Bowl RNAi (*F*,*F*′), stained for Sens (red). Note the cell-autonomous loss of Sens expression in *hyd*^15^ mutant clones and the recovery of Sens expression upon Bowl RNAi (cf. arrowheads). (*G–H*′) A third instar eye disc containing GFP-positive MARCM clones of *hyd*^*15*^ (*G*,*G*′) or *hyd*^*15*^ with Bowl RNAi (*H,H*′), stained for Elav (red) to label the photoreceptors. Anterior is to the *left*, and arrows mark the morphogenetic furrow (MF). Elav-positive photoreceptors are normally detected only posterior to the MF. *hyd*^15^ mutant clones, especially those close to the MF, showed ectopic Elav-positive photoreceptors anterior to the MF, and this phenotype was rescued by Bowl RNAi (cf. arrowheads). (*I–J*′) Similar to *E–F*′ except that *lin*^*A25*^ mutant clones without (*I*,*I*′) or with (*J*,*J*′) Bowl RNAi were analyzed. Note the cell-autonomous loss of Sens expression in *lin*^*A25*^ mutant clones and the recovery of Sens expression upon Bowl RNAi (cf. arrowheads). (*K–L*′) Similar to *G–H*′ except that *lin*^*A25*^ mutant clones without (*K*,*K*′) or with (*L*,*L*′) Bowl RNAi were analyzed. *lin*^*A25*^ mutant clones, especially those close to the MF, showed ectopic Elav-positive photoreceptors anterior to the MF, and this phenotype was rescued by Bowl RNAi (cf. arrowheads).

Hyd was one of the first tumor suppressor genes isolated in *Drosophila* (Martin et al. 1977; Watson et al. 1994). However, the direct substrate of Hyd that accounts for its tumor suppressor activity has remained elusive. Our identification of Hyd as an E3 ubiquitin ligase for Bowl degradation therefore uncovers the elusive substrate for this orphan tumor suppressor. If this is the case, one would expect *hyd* mutant cells to accumulate Bowl protein in vivo, as we observed in *lin* mutant cells. Indeed, *hyd* mutant clones showed a robust, cell-autonomous, increase of Bowl protein level, as predicted by our model (Fig. 5A,A′). Besides Bowl accumulation, *hyd* mutant clones also resembled *lin* mutant clones in clonal overgrowth, round shape of the mutant clones, and upregulation of Dl expression (Fig. 5A–B′). Importantly, all these phenotypes were completely rescued by RNAi knockdown of Bowl, implicating Bowl as a critical downstream effector of Hyd (Fig. 5C–D′).

Previous genetic studies in *Drosophila* have reported complex phenotypes for *hyd* in imaginal disc development. For example, Hyd was reported to be required for the expression of the proneural gene *senseless* (*sens*) along the dorsal/ventral (D/V) boundary of the wing imaginal disc (Flack et al. 2017). On the other hand, Hyd was shown to repress neural development in the eye imaginal disc, with loss of *hyd* leading to precocious photoreceptor differentiation anterior to the morphogenetic furrow (MF) (Lee et al. 2002). We wondered whether these complex phenotypes may also depend on the accumulation of Bowl in *hyd* mutant clones. Strikingly, RNAi knockdown of Bowl fully rescued the loss of *sens* expression in the wing disc (Fig. 5E–F′) and the precocious photoreceptor differentiation in the eye disc (Fig. 5G–H′) associated with *hyd* mutant clones. As in *hyd* mutant clones, *lin*^*A25*^ mutant clones caused a similar loss of *sens* expression in the wing disc and precocious photoreceptor differentiation in the eye disc, both of which were completely rescued by RNAi knockdown of Bowl (Fig. 5I–L′). These genetic epistasis results further support our model implicating Bowl as a critical physiological substrate of the Hyd–Lin E3 ligase complex.

### The Hyd–Lin–Bowl pathway couples the Polycomb repressive complex 1 to tissue growth in *Drosophila*

**Figure 6.**
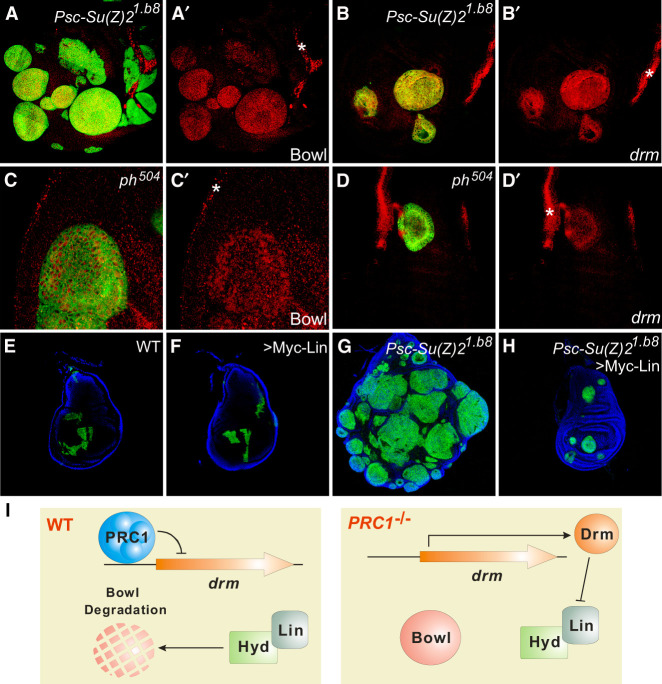
The Hyd–Lin–Bowl pathway is regulated by PRC1 through epigenetic silencing of *drm* expression. (*A–B*′) A third instar wing disc containing GFP-positive MARCM clones of *Psc-Su(Z)*2^*1.b8*^, stained for Bowl protein (red, *A*,*A*′) or analyzed for *drm* mRNA expression by FISH (red, *B*,*B*′). Note the cell-autonomous increase of Bowl protein level and mRNA level of *drm* in the mutant clones. Also note that Bowl and *drm* are normally preferentially expressed around the disc periphery (asterisks) but much less in the center of the wing disc. (*C–D*′) A third instar wing disc containing GFP-positive MARCM clones of *ph*^*504*^, stained for Bowl protein (red, *C*,*C*′) or analyzed for *drm* mRNA expression by FISH (red, *D*,*D*′). Note the cell-autonomous increase of Bowl protein level and mRNA level of *drm* in the mutant clones. Asterisks mark the peripheral zone with high normal Bowl and *drm* expression, as in *A–B*′. (*E–H*) A third instar wing disc containing mutant clones of the indicated genotype, generated by induction of MARCM clones during the first instar stage. The clones are marked by GFP expression (green), and the discs were counterstained with DAPI (blue). Note the similar size of MARCM clones overexpressing a Myc-tagged Lin transgene (*F*) compared with the wild-type control clones (*E*). Also note that the massive overgrowth of *Psc-Su(Z)2*^*1.b8*^ mutant clones (*G*) was significantly suppressed by the expression of the Myc-Lin transgene (*H*). Quantification of clone size is shown in Supplemental Figure S6D. (*I*) A schematic model describing the PRC1–Hyd pathway. (*Left*) PRC1 normally silences *drm* expression, which in turn allows Hyd–Lin to degrade Bowl, thus restricting tissue growth. (*Right*) Loss of PRC1 leads to derepression of *drm* expression. Drm then relieves Bowl from Hyd–Lin-mediated degradation, resulting in Bowl stabilization and tissue overgrowth.

After establishing the Hyd–Lin–Bowl pathway in growth control, we next investigated whether and how this pathway is regulated in developing tissues. We hypothesized that if an upstream regulator exists, its genetic perturbation may also affect Bowl protein level. We therefore surveyed known signaling pathways and growth regulators for their effect on Bowl protein accumulation. Although increased activities of Yki, Ras, PI3K, Dpp, Notch, Wnt, JAK/STAT, and Hh signaling had no effect on Bowl protein level (Supplemental Fig. S5), mutations in *Psc-Su(Z)2* and *ph*, which encode different subunits of the epigenetic regulator Polycomb repressive complex 1 (PRC1), resulted in cell-autonomous accumulation of Bowl protein in mutant clones (Fig. 6A,A′,C,C′), suggesting that the Hyd–Lin–Bowl pathway may be regulated by this repressive chromatin modifier. In contrast, no Bowl accumulation was observed in mutant clones of Polycomb repressive complex 2 (PRC2) components *E(z)* or *Su(z)12* (Supplemental Fig. S5I–J′), highlighting the specificity of Bowl accumulation in PRC1 mutant clones. Notably, PRC1 (but not PRC2) has been shown to function as a tumor suppressor in *Drosophila* imaginal discs, with its loss of function leading to tissue overgrowth (Beuchle et al. 2001; Oktaba et al. 2008; Classen et al. 2009; Martinez et al. 2009).

Given the well-established role for the Polycomb repressive complexes in silencing developmental genes through histone modification (Entrevan et al. 2016), we first examined whether *bowl* transcription is normally silenced by PRC1. However, despite the increased Bowl protein level, *bowl* mRNA level was not elevated in PRC1 mutant clones (Supplemental Fig. S6A–B′). This prompted us to examine the possibility that PRC1 represses the transcription of Drm, the micropeptide/pseudosubstrate inhibitor for Hyd–Lin-mediated Bowl degradation (Fig. 6I). Consistent with this hypothesis, we noted that in genome-wide profiling of PRC binding, including those conducted in *Drosophila* cell lines, embryos, and imaginal discs (Schwartz et al. 2006; Tolhuis et al. 2006; Schuettengruber et al. 2014; Loubiere et al. 2016), the *drm* promoter is enriched for PRC1 binding sites (Supplemental Fig. S6C, for example). Indeed, using fluorescent in situ hybridization (FISH), we found that the mRNA level of *drm* was markedly increased cell-autonomously in PRC1 mutant clones (Fig. 6B,B′,D,D′). These results suggest that loss of PRC1 leads to cell-autonomous induction of the micropeptide Drm, which in turn functions as a pseudosubstrate to inhibit Lin function and therefore stabilizes Bowl. In agreement with this model, we found that overexpression of Lin, which did not affect the growth of wild-type control clones, greatly suppressed the overgrowth of PRC1 mutant clones (Fig. 6E–H; Supplemental Fig. S6D). These findings implicate the Hyd–Lin–Bowl pathway as an important downstream effector of PRC1 in growth control (Fig. 6I).

### Functional conservation of the Hyd–Lin–Bowl pathway in human cells

After elucidating the Hyd–Lin-mediated Bowl degradation pathway in *Drosophila*, we next explored whether a similar pathway also operates in human cells. To this end, we transiently expressed *Drosophila* Lin and a minimal Bowl fragment that is susceptible to Lin-induced degradation (Bowl^122–373^) in HEK293 cells and found that Lin also decreased the protein level of Bowl (Supplemental Fig. S7A), suggesting that a Hyd-like activity is present in this cell line. Lin expression similarly decreased the protein level of coexpressed OSR1 or OSR2, two human homologs of Bowl (Supplemental Fig. S7A), implying OSR1 and OSR2 as potential substrates of this Hyd-like activity in HEK293 cells.

**Figure 7.**
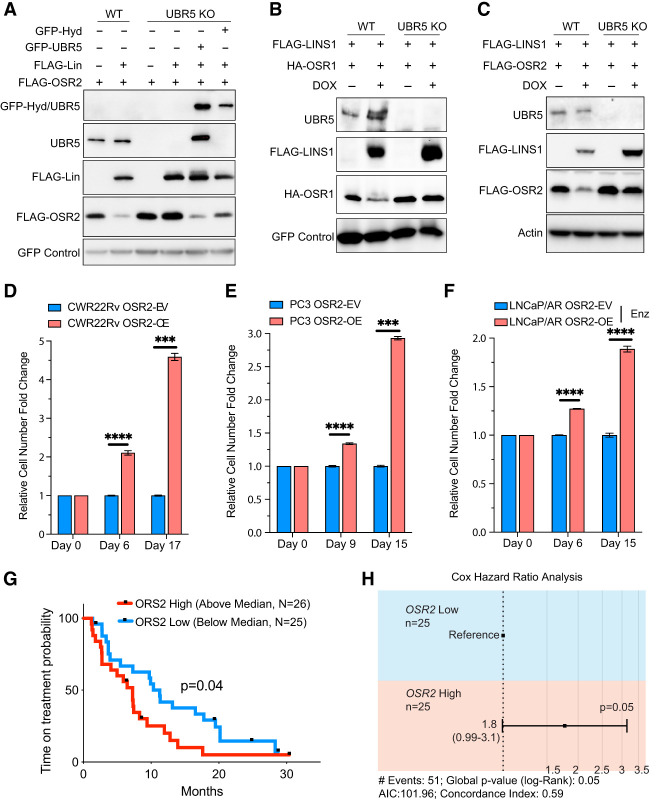
Functional conservation of the UBR5–LINS1–OSR1/2 axis in human cells. (*A*) HEK293 cell lysates expressing the indicated constructs were analyzed by Western blotting. Note the dramatic reduction of FLAG-OSR2 when coexpressed with FLAG-Lin in wild-type, but not UBR5 knockout, cells. Also note that Lin-induced decrease of FLAG-OSR2 was restored by reintroduction of GFP-Hyd or GFP-UBR5 in UBR5 knockout cells. (*B*,*C*) HEK293 cell lysates expressing the indicated constructs were analyzed by Western blotting. Note the dramatic reduction of HA-OSR1 (*B*) or HA-OSR2 (*C*) when coexpressed with FLAG-LINS1 in wild-type, but not UBR5 knockout, cells. (*D*) Relative cell number fold change of CWR22Rv cells transduced with annotated constructs, normalized to the OSR2-EV (empty vector) group, measured in a FACS-based competition assay. (OSR2-OE) OSR2 overexpression. *P*-values were calculated using multiple *t*-tests (*n* = 3; mean ± SEM). (*E*) Relative cell number fold change of PC3 cells transduced with annotated constructs, normalized to the OSR2-EV (empty vector) group, measured in a FACS-based competition assay. (OSR2-OE) OSR2 overexpression. *P*-values were calculated using multiple *t*-tests (*n* = 3; mean ± SEM). (*F*) Relative cell number fold change of LNCaP/androgen receptor AR) cells transduced with annotated constructs, normalized to the OSR2-EV (empty vector) group, measured in a FACS-based competition assay. (Enz) Enzalutamide (10 µM) treatment medium supplemented with charcoal-stripped serum, (OSR2-OE) OSR2 overexpression. *P-*values were calculated using multiple *t*-tests (*n* = 3; mean ± SEM). (*G*) Progression-free survival on AR targeted therapies (enzalutamide or abiraterone) of patients with high (above median) or low (below median) expression of OSR2 of the Stand Up to Cancer (SU2C) cohort. *P*-value was calculated using the log rank (Mantel–Cox) test. (*H*) Cox hazard ratio analysis of the patients with high (above median) or low (below median) expression of OSR2 of the SU2C cohort. *P*-value was calculated using the log rank (Mantel-Cox) test. For all panels, mean ± SEM is represented. (****) *P* < 0.0001, (***) *P* < 0.001.

The human homolog of Hyd is UBR5. Consistent with UBR5 as an Hyd-like E3 ligase in mammalian cells, RNAi knockdown of UBR5 in HEK293 cells suppressed Lin-induced downregulation of Bowl, OSR1, or OSR2 (Supplemental Fig. S7B–D). To further corroborate these results, we deleted UBR5 in HEK293 cells by CRISPR/Cas9. Consistent with the UBR5 RNAi results, Lin was unable to decrease the protein level of Bowl (Supplemental Fig. S7E) or OSR2 (Fig. 7A) in UBR5 knockout cells. This defect was rescued by reintroduction of UBR5 or Hyd in UBR5 knockout cells (Fig. 7A; Supplemental Fig. S7E). Together, these results implicate UBR5 as a functional homolog of Hyd in mammalian cells.

Next, we tested LINS1, the human homolog of Lin. Unlike OSR1/2 and UBR1, we could not detect the expression of epitope-tagged LINS1 using the generic pcDNA vector. We therefore turned to a doxycycline (Dox)-inducible vector to express LINS1. Indeed, Dox-induced expression of LINS1 markedly decreased the protein level of coexpressed OSR1 (Fig. 7B) or OSR2 (Fig. 7C) in wild-type but not UBR5 knockout cells. Thus, as their *Drosophila* counterpart, LINS1 decreases the protein level of OSR1/2 in human cells in a UBR5-dependent manner, suggesting that the Hyd–Lin–Bowl pathway is conserved in human cells.

As a starting point to functionally interrogate this pathway in human cells, we investigated whether overexpression of OSR2 (akin to its *Drosophila* homolog, Bowl) promotes cell proliferation and tumorigenesis. Indeed, overexpression of OSR2 enhanced the proliferation of HEK293T cells as measured by a FACS-based competition assay (Supplemental Fig. S7F–H). Given that genomic amplification of OSR2 occurs in as many as 30% of prostate cancer patients (Supplemental Fig. S7I; Grasso et al. 2012; The Cancer Genome Atlas Research Network 2015; Kumar et al. 2016; Armenia et al. 2018; Ren et al. 2018; Abida et al. 2019), we next examined the impact of OSR2 overexpression on the proliferation of prostate cancer cells. Remarkably, OSR2 overexpression in two advanced prostate cancer cell lines, CWR22Rv and PC3, significantly enhanced the growth of these tumor cells (Fig. 7D,E). Like its *Drosophila* counterpart, Bowl, OSR2 overexpression also activates Notch signaling, as indicated by the elevated expression of multiple Notch target genes in CWR22Rv and PC3 cells (Supplemental Fig. S7J,K). Interestingly, previous studies have shown that activation of Notch signaling contributes to the development of androgen receptor (AR) independence and resistance to antiandrogens in prostate cancer (Stoyanova et al. 2016; Farah et al. 2019). We therefore explored a potential role for OSR2 in modulating AR dependence and responses to antiandrogens such as enzalutamide in the LNCaP/AR cell line, a well-credentialed AR-dependent model that is sensitive to enzalutamide. Supporting this hypothesis, OSR2 overexpression in LNCaP/AR cells not only induced the expression of multiple Notch target genes (Supplemental Fig. S7L) but also conferred growth advantage in enzalutamide-containing media (Fig. 7F). This hypothesis gains further support from the analysis of an advanced prostate cancer genomic study, the Stand Up to Cancer (SU2C) cohort, in conjunction with longitudinal clinical outcome data (Abida et al. 2019; Zhang et al. 2020b). Our analysis demonstrated that patients exhibiting high levels of OSR2 expression (above median expression) experience a significantly shorter time to progression on antiandrogens compared with those with low OSR2 expression (below median expression) (Fig. 7G). Cox hazard ratio analysis corroborated this finding, revealing increased risks of antiandrogen-resistant tumor progression associated with elevated OSR2 expression (Fig. 7H). Taken together, these results implicate a growth-promoting function of OSR2 similar to that of its *Drosophila* counterpart.

## Discussion

In this study, we delineate a novel tumor suppressor pathway that links epigenetic program to regulated protein degradation in tissue growth control. A key component of this pathway is the E3 ubiquitin ligase Hyd, which, through the adaptor protein Lin, recognizes and ubiquitinates a critical substrate, Bowl. Conversely, the micropeptide Drm, whose expression is normally repressed by PRC1, functions as a pseudosubstrate that stabilizes Bowl by competing Bowl off the Lin–Hyd E3 ligase complex (Fig. 6I). Thus, when Lin or Hyd is compromised, Bowl is stabilized and drives tissue overgrowth. Loss of PRC1 results in a similar accumulation of Bowl due to induction of Drm expression (Fig. 6I). Our findings that a similar mechanism operates in human cells where UBR5 acts together with LINS1 to decrease the protein level of OSR1/2, together with the growth-promoting function of OSR2 in mammalian cells, suggest that further investigation of this pathway in mammalian physiology and disease is warranted.

The elucidation of the PRC1-regulated Hyd–Lin–Bowl pathway bridges several gaps in our understanding of growth control. First, by identifying Bowl as the long sought-after physiological substrate of Hyd, we have elucidated the molecular function of this orphan tumor suppressor, demonstrating that its developmental function can be largely accounted for by its role in Bowl degradation. These findings therefore provide a unified explanation for the complex mutant phenotypes of this enigmatic tumor suppressor. Second, through the analysis of hypomorphic alleles, we have uncovered a hidden function of *lin* as a tumor suppressor gene that would otherwise be masked by the pleiotropic effect of a strong/null allele, akin to several gene dosage-dependent tumor suppressors such as ATR (Fang et al. 2004), HDAC1/2 (Heideman et al. 2013), and Dicer (Kumar et al. 2009). Third, our characterization of Lin as a Hyd-dependent substrate adaptor protein resolves the biochemical mechanism of how Lin destabilizes Bowl. This conclusion is supported not only by biochemical studies linking Lin and Hyd in a common pathway of Bowl degradation but also genetic studies demonstrating the similarities of *hyd* and *lin* mutant phenotypes and their dependency on elevated Bowl activity. Last, our identification of PRC1 as a transcriptional repressor of Drm implicates the Drm–Hyd–Lin–Bowl cascade as an important tumor suppressor pathway that couples this critical epigenetic regulator to tissue growth and tumorigenesis (Fig. 6I). Whether the Drm–Hyd–Lin–Bowl cascade is regulated by additional upstream inputs besides PRC1 and how the zinc finger protein Bowl regulates the expression of downstream genes such as Dl remain to be elucidated.

The human homolog of Hyd, UBR5, is recurrently dysregulated in many cancer types, but how UBR5 dysregulation contributes to tumorigenesis remains poorly defined (Shearer et al. 2015). Although multiple targets of UBR5 have been reported, such as the deubiquitinase DUBA (Rutz et al. 2015), the E3 ligase RNF168 (Gudjonsson et al. 2012; Okamoto et al. 2013), c-Myc and its cooperative transcriptional factors (Mark et al. 2023), and nuclear hormone receptors (Tsai et al. 2023), the role of these targets in UBR5-associated tumors is unclear. Interestingly, in none of these situations was UBR5 shown to engage its respective substrate through a substrate adaptor. Therefore, our study represents the first identification of a substrate adaptor for the UBR5 ubiquitin ligases. Another striking finding from our study is that the developmental function of Hyd/UBR5 can be largely accounted for by a single substrate, Bowl, in *Drosophila*. In a similar fashion, the developmental function of glycogen synthase kinase 3 (GSK-3) and cAMP-dependent protein kinase (PKA), both of which are pervasive enzymes with numerous biochemical substrates, can be attributed to specific substrates in Wnt and Hedgehog signaling: β-catenin (Rim et al. 2022) and Ci/Gli (Zhang and Beachy 2023), respectively. The design principles underlying such exquisite specificity in developmental signaling are worth further investigation.

## Materials and methods

### Cell line

HEK293 and HEK293T cells were cultured in DMEM supplemented with 10% fetal bovine serum (FBS), 1% L-glutamine, and 1% penicillin–streptomycin at 37°C in a humidified atmosphere with 5% CO_2_. LNCaP/AR, CWR22Rv, and PC3 cells were cultured in RPMI 1640 medium supplemented with 10% FBS, 1% L-glutamine, 1% penicillin–streptomycin, 1% HEPES, and 1% sodium pyruvate at 37°C in a humidified atmosphere with 5% CO_2_. S2R^+^ cells were cultured in *Drosophila* Schneider's medium supplemented with 10% FBS (Gibco) and antibiotics at 25°C in a humidified atmosphere.

### *Drosophila* genetics

Flies were reared on standard cornmeal, molasses, and yeast medium at 25°C and 50% humidity and maintained on a 12 h:12 h light:dark cycle. The following stocks were obtained from Bloomington *Drosophila* Stock Center: *lin*^*G2*^ (#7087), *lin*^*2*^ (#3099), *bowl*^*1*^ (#7094), *hyd*^*15*^ (#3718), *Psc-Su(Z)2*^*1.b8*^ (#24467), *ph*^*504*^ (#24162), *ptc*^*S2*^ (#6332), *E(z)*^*731*^ (#24470), *Su(z)12*^*4*^ (#24469), *UAS-bowl*^*RNAi*^ (#34735), *UAS-lin* (#7074), *UAS-PI3K* (#25915), *UAS-Ras*^*V12*^ (#64195), *UAS-Arm*^*S10*^ (#4782), and *dpp-lacZ* (#12379). The following stocks were obtained from Vienna *Drosophila* Resource Center: *UAS-bowl*^*RNAi*^ (#3774 and #102050), *UAS-Dl*^*RNAi*^ (#37287, #37288, and #109491), and *VT044106-Gal4* (VT44106). The *P[lin+]* genomic rescue vector was constructed by cloning the genomic DNA of the *lin* locus (8,912,607; 8,918,526) into pattB, and the transgene was landed on an 86Fa attP site. An enhancer trap, *Dl-lacZ*, was obtained from Bloomington *Drosophila* Stock Center (#11651). A *Dl-lacZ* transgenic reporter on other chromosomes was constructed by cloning the genomic enhancer of *Dl* (19,312,938; 19,315,041) into the pCplzN vector. The *UAS-HA-Bowl*, *UAS-HA-Bowl*^*K137R*^, and *UAS-Myc-Lin* fly lines were also made in our laboratory. The following flies have been described previously: *10XSTAT92E-GFP* (Bach et al. 2007), *GbeSu(H)-lacZ* (gift of Dr. Sarah Bray; Furriols and Bray 2001), *UAS-yki* (Huang et al. 2005), *UAS-tkv*^*Act*^ (Hoodless et al. 1996), *UAS-N*^*icd*^ (Cooper and Bray 2000), and *UAS-upd* (Classen et al. 2009).

For all experiments involving *bowl*^*RNAi*^ and *Dl*^*RNAi*^, multiple *UAS-RNAi* lines were tested, and all gave similar results. All crosses were done at 25°C.

Mitotic recombination clones were generated by FLP-*FRT* recombination (Xu and Rubin 1993). Clones in adult eyes were generated using the eyeless-FLP/recessive cell-lethal technique (Newsome et al. 2000) in flies of the following genotypes: *y w eyFlp GMR-lacZ/+; FRT42D/FRT42D l(2)c1-R11 P[w+]*, *y w eyFlp GMR-lacZ/+; FRT42D lin^A24^/FRT42D l(2)c1-R11 P[w+]*, *y w eyFlp GMR-lacZ/+; FRT42D lin^A25^/FRT42D l(2)c1-R11 P[w+]*, and *y w eyFlp GMR-lacZ/+; FRT42D lin^G2^/FRT42D l(2)c1-R11 P[w+]*.

MARCM (mosaic analysis with a repressible cell marker) clones were generated as described by Lee and Luo (1999) in flies of the following genotypes: control MARCM clones (*hsFlp UAS-GFP/+; FRT42D/FRT42D tub-Gal80; tub-Gal4/+*), *lin* mutant MARCM clones (*hsFlp UAS-GFP/+; FRT42D lin^A24^/FRT42D tub-Gal80; tub-Gal4/+*, *hsFlp UAS-GFP/+; FRT42D lin^A25^/FRT42D tub-Gal80; tub-Gal4/+*, *hsFlp UAS-GFP/+; FRT42D lin^G2^/FRT42D tub-Gal80; tub-Gal4/+*, *hsFlp UAS-GFP/+; FRT42D lin^A24^/FRT42D tub-Gal80; tub-Gal4/P[lin+]*, *hsFlp UAS-GFP/+; FRT42D lin^A25^/FRT42D tub-Gal80; tub-Gal4/ P[lin+]*, *hsFlp UAS-GFP/+; FRT42D lin^G2^/FRT42D tub-Gal80; tub-Gal4/ P[lin+]*, *hsflp UAS-GFP/+; FRT42D lin^A25^/FRT42D tub-Gal80; tub-Gal4/UAS-bowl*^*RNAi*^, *hsflp UAS-GFP/+; FRT42D lin*^*A25*^*/FRT42D tub-Gal80; tub-Gal4/UAS-Dl*^*RNAi*^, *hsflp UAS-GFP/+; FRT42D lin^A25^/FRT42D tub-Gal80; tub-Gal4/Dl-lacZ*, and *hsflp UAS-GFP/+; FRT42D lin^A25^/FRT42D tub-Gal80; tub-Gal4/GbeSu(H)-lacZ/+*), *hyd*^*15*^ mutant MARCM clones (*hsFlp UAS-GFP tub-Gal4/+; FRT82B hyd^15^/FRT82B tub-Gal80* and *hsFlp UAS-GFP tub-Gal4/+; UAS-bowl^RNAi^/+; FRT82B hyd^15^/FRT82B tub-Gal80*), *Psc-Su(Z)2*^*1.b8*^ or *ph*^*504*^ mutant MARCM clones (*hsFlp UAS-GFP/+; FRT42D Psc-Su(Z)2^1.b8^/FRT42D tub-Gal80; tub-Gal4/+*, *hsFlp UAS-GFP/+; FRT42D Psc-Su(Z)2^1.b8^/FRT42D tub-Gal80; tub-Gal4/UAS-Myc-Lin*, and *19A ph^504^/19A tub-Gal80 hs-flp; UAS-GFP/+; tub-Gal4/+*), *Dl* RNAi MARCM clones (*hsFlp UAS-GFP/+; FRT42D/FRT42D tub-Gal80; tub-Gal4/UAS-Dl*^*RNAi*^), Bowl-overexpressing MARCM clones (*hsFlp UAS-GFP/+; FRT42D/FRT42D tub-Gal80; tub-Gal4/UAS-Bowl*), and Lin-overexpressing MARCM clones (*hsFlp UAS-GFP/+; FRT42D/FRT42D tub-Gal80; tub-Gal4/UAS-Myc-Lin*).

FLP-out clones were generated using the transgene *Act>CD2>Gal4* (Pignoni and Zipursky 1997). The following genotypes were used: Bowl-overexpressing FLP-out clones (*hsFlp; act>>Gal4 UAS-GFP/+; UAS-HA-Bowl/+*) and Dl-overexpressing Flp-out clones (*hsFlp; act>>Gal4 UAS-GFP/UAS-Dl*).

### Immunostaining and antibodies

Imaginal discs were fixed and stained following standard formaldehyde fixation and permeabilization/washes in PBT/0.3% Triton X-100. Bowl antibodies were produced by immunizing rabbits with the APPRRTGFSIEDIMRR peptide (Pierce), and the antisera were used at 1:500 dilution for immunostaining. The following antibodies were also used for immunostaining: mouse anti-Myc (clone 9E10, 1:200; Millipore Sigma 05-419), rabbit anti-HA (1:200; Cell Signaling 3724), mouse anti-β-Gal (1:100; Developmental Studies Hybridoma Bank [DSHB] JIE7), mouse anti-Dl (1:300; DSHB C594.9B), guinea pig antisenseless (1:1000; gift from Hugo Bellen), and rat anti-Elav (1:200; DSHB 7E8A10).

The following antibodies were used for Western blot assay: rabbit polyclonal anti-UBR5 antibody (1:1000; ABclonal A13816), mouse anti-Myc antibody (1:1000; Millipore Sigma 05-419), mouse anti-FLAG antibody (1:1000; Sigma-Aldrich A8592), mouse anti-HA antibody (1:1000; Sigma-Aldrich 11583816001), mouse antiubiquitin antibody (1:100; Santa Cruz Biotechnology sc-8017), mouse antiactin (1:10,000; Millipore Sigma MAB1501R), rabbit antitubulin (Cell Signaling Technology 2148S), and rabbit anti-GFP (1:1000; Cell Signaling Technology 2555S).

### Fluorescent in situ hybridization

Fluorescent RNA probes for *drm*, *sob*, *odd*, and *bowl* were custom-designed and synthesized by LGC Biosearch Technologies. In situ hybridization was done according to the manufacture's protocol with minor modifications. Briefly, imaginal discs were dissected in PBS and fixed in 4% paraformaldehyde for 45 min at room temperature. The samples were then washed twice with PBS and permeabilized in 70% ethanol overnight at 4°C. The discs were then pretreated with wash buffer A for 5 min at room temperature, followed by incubation in hybridization buffer in Eppendorf tubes for 4 h at 37°C. The samples were then treated with wash buffer A, stained with DAPI, washed with wash buffer B, and mounted on slides.

### Plasmids

Bowl (LD15350), Lin (LD43682), and Drm (LD26791) cDNAs were obtained from the *Drosophila* Genomics Resource Center. HA-tagged Bowl and Drm and FLAG- and Myc-tagged Lin were cloned into pAC5.1/V5-HisB by In-Fusion (Clontech). Hyd-HA was a gift from Shi'an Wu. Site-directed mutagenesis was used to generate mutant Lin (A24 and A25) or Bowl (K137R and/or K139R) protein. Human cDNA for LINS1, OSR1, and OSR2 was cloned from HEK293 cells by RT-PCR and verified by DNA sequencing. The DNA fragments for OSR1 and OSR2 were cloned into pCDH-CMV vector (EcoRI/NotI). The DNA fragments for LINS1 were cloned into pENRT4-FLAG using NEBuilder HiFi DNA assembly master mix. LINS1 was further cloned into pCW57.1 by Gateway cloning. GFP-UBR5 was from Addgene (52050).

### Cell culture and RNAi

*Drosophila* S2R^+^ cells were cultured in *Drosophila* Schneider's medium supplemented with 10% FBS (Gibco) and antibiotics at 25°C. Transfection, immunoprecipitation, and Western blotting were carried out as described previously (Yu et al. 2010). For the dsRNA screen, the DNA templates of dsRNAs from *Drosophila* RNAi library (Open Biosystem) were PCR-amplified and transcribed with 5XMEGAScript T7 kit (Ambion). The list of 179 E3 ubiquitin ligases in the dsRNA library, including the dsRNA targeting sequences, and additional Hyd dsRNA targeting sequences are shown in Supplemental Table S1. Briefly, on the first day, S2R^+^ cells grown in 100 mm plates at ∼70% confluence were transfected with pAC-FLAG-Lin and pAC-HA-Bowl^122–373^. The next day, the cells were split into 24 well plates with 2 × 10^4^ cells/well. On the third day, each well was treated with four individual dsRNAs (1 μg each) using the bathing protocol (http://fgr.hms.harvard.edu/protocols). On day 7, samples were collected by lysis in the 2× SDS-PAGE loading buffer.

HEK293 cells were cultured in DMEM (Invitrogen) supplemented with 10% FBS (Gibco) and antibiotics at 37°C. Pooled UBR5 siRNAs (L-007189-00-0005) were purchased from Dharmacon. Wild-type or UBR5 knockout HEK293 cells were seeded in a 12 well plate at a confluency of 50%–60%. Plasmid transfection was conducted using FuGENE HD transfection reagent. To induce LINS1 expression, culture medium was replaced with fresh medium containing 200 ng/mL doxycycline after 12 h of transfection. For the knockdown assay, 10 nM siRNA was transfected 12 h before plasmid transfection using Lipofectamine RNAiMAX transfection reagent. All transfections were conducted according to the manufacturer's instructions.

Parental LNCaP/AR, CWR22Rv, and HEK293T cell lines were obtained from Charles Sawyers’ laboratory at Memorial Sloan Kettering Cancer Center, and PC3 (CRL-1435) cell lines were purchased from ATCC. LNCaP/AR, CWR22Rv, and PC3 cells were cultured in RPMI 1640 medium supplemented with 10% FBS, 1% L-glutamine, 1% penicillin–streptomycin, 1% HEPES, and 1% sodium pyruvate. HEK293T cells were cultured in DMEM supplemented with 10% FBS, 1% L-glutamine, and 1% penicillin–streptomycin. LNCaP/AR cells were cultured in RPMI 1640 medium supplemented with 10% charcoal-stripped serum (CSS) medium when treated with 10 µM Enz. LNCaP/AR, PC3, and HEK293T cells were passaged at a 1:6 ratio every 3–5 days. CWR22Rv cells were passaged at 1:3 ratio every 3–5 days. Cell cultures were assessed for mycoplasma via MycoAlert Plus mycoplasma detection kit (Lonza LT07-710) monthly, and all results were negative. STR profiling cell authentication was used to validate cell line identification, compared with ATCC profiles, every year.

### Knockout cell line generation

The CRISPR/Cas9 approach was used to generate the UBR5 knockout HEK293T cell line. The gRNA sequence (5′-CACCGTATGATTATGTTTGGGTCGC-3′) was designed by a web tool (Cong et al. 2013), and the DNA oligos were annealed and ligated into pSpCas9n(BB)-2A-Puro vector (BbsI/BbsI sites). Assembled gRNA sequences were validated by Sanger sequencing. HEK293T cell transfection was carried out in a 6 cm dish at a confluency of ∼60% with 5 μg of pSpCas9n(BB)-2A-Puro-gRNA plasmid using Lipofectamine 3000 transfection reagent according to the manufacturer's instructions. Twenty-four hours after transfection, 1 μg/mL puromycin was added to the medium for positive selection for 2 days. One-thousand positive cells were replated on 15 cm dishes. One week later, single clones were picked to expand by transferring into 48 well plates and then 12 well plates. Selected clones were confirmed by PCR and validated by Sanger sequencing and Western blotting with anti-UBR5 antibodies. The specific UBR5 knockout clone used for further analysis was transheterozygous with two mutations: a 6 bp deletion, resulting in a stop codon immediately after Gly1346, and a 13 bp sequence replaced by a 16 bp sequence, resulting in a truncated protein of 1344 amino acids. Both mutations are predicted to truncate UBR5 before the enzymatically critical HECT domain: wild type (GAAATCTATGATTATGTTTGGGTC-GCAGGAGAATAAAGA), mutation#1 (GAAATCTATGATTATGTTTGGGTAGAATAAAGA), and mutation#2 (GAAATCTATGAATCTATGATTCTTCCTGCAGGAGAATAAAGA).

### Ubiquitination assay

Detection of Bowl protein ubiquitination was carried out according to Choo and Zhang (2009). Briefly, S2R^+^ cells treated with 10 μM proteasome inhibitor PS-341 for 5 h were collected by centrifugation and then lysed in boiling lysis buffer (2% SDS, 150 mM NaCl, 10 mM Tris-HCl at pH 8.0) for 10 min. DNA was sheared by sonication, and cell extracts were diluted by 10-fold with dilution buffer (10 mM Tris-HCl at pH 8.0, 150 mM NaCl, 2 mM EDTA, 1% Triton) followed by 1 h of incubation with rotation at 4°C. Cell extracts were cleared by centrifugation at 13,000 rpm for 10 min and the supernatant was incubated with anti-FLAG agarose affinity gel (Sigma A4596) overnight with rotation at 4°C. Beads were washed three times in washing buffer (10 mM Tris-HCl at pH 8.0, 1 M NaCl, 1 mM EDTA, 1% NP-40) and then boiled in 2× SDS buffer for 5 min. Proteins were separated on a precast 4%–20% SDS-PAGE gradient gel (Bio-Rad) and detected by mouse antiubiquitin antibodies (Santa Cruz Biotechnology sc-8017 HRP).

### Gene expression detection by qPCR and Western blot

Total RNA from cells was extracted using Trizol (Ambion 15596018), and cDNA was made using the SuperScript IV Vilo master mix with ezDNase enzyme (Thermo Fisher 11766500) with a 200 ng/µL RNA template. cDNA was amplified with 2×PowerUp SYBR Green master mix (Thermo Fisher A25778). Only HES/HEY family genes with cycle threshold (CT) values >30 in qPCR were quantified for relative expression. For Western blots, proteins were extracted from cell lysate using RIPA buffer and then measured with the Pierce BCA protein assay kit (23225). Protein lyses were boiled for 5 minutes at 95°C and run on NuPAGE 4%–12% Bis-Tris gels (Invitrogen NP0323). Transfer was conducted for 1 h at 100 V at 4°C. Membranes were then blocked for 15 min in 5% nonfat milk prior to incubation with primary antibody and then washed with 1× TBST (10× stock from Teknova T9511).

### FACS-based competition assay

FACS-based competition assay was performed as previously described (Zhang et al. 2020b). Specifically, the OSR2-EV and OSR2-OE cells were transduced with pLKO5.sgRNA.EFS.GFP and pLKO5.sgRNA.EFS.RFP to express fluorescent color. Next, the OSR2-EV (RFP) and OSR2-OE (GFP) cells were mixed into a cell mixture, and the percentage of RFP-positive cells was measured on various days by FACS. The LNCaP/AR cell mixture was treated with CSS medium and 10 μM Enz during the competition experiment. Cells were first gated based on SSC-H/FSC-A → FSC-H before measuring the RFP/GFP signals. Relative cell number fold change was calculated and normalized to the veh-treated group as previously described (Zhang et al. 2020b). Three biological triplicates were used, mean ± SEM was reported, and experiments were repeated at least twice and achieved similar conclusions. No data points were excluded. Attune Nxt (version 4.2.1627.1) and FlowJo (version 10.8.0) were used for FACS data analysis.

### Human prostate cancer data analysis

The frequency of OSR2 alterations in various prostate cancer patient cohorts was acquired through http://www.cbioportal.org. Processed 444 Stand Up To Cancer (SU2C) metastatic prostate cancer patient cohort (Abida et al. 2019) RNA-seq data and enzalutamide/abiraterone treatment data were downloaded from http://www.cbioportal.org. Twenty-nine patients of this cohort were excluded because they had SPOP mutations, which demonstrate elevated sensitivity to antiandrogen treatment (Boysen et al. 2018). Only a subset of patients within this cohort (*n* = 51) had baseline biopsies, poly(A) RNA-seq results, and matched clinical data, which were used for progression-free survival and Cox hazard analyses. The Kaplan–Meier plot for progression-free survival was generated by Prism 9 using the Mantel–Cox test. Cox hazard ratio analysis was performed using the R package “survminer.”

### Quantification and statistical analysis

AxioVision 4.8 and ZEN 3.8 were used to measure clone and compartment size in wing imaginal discs. Mean fluorescence intensity was measured using ZEN 3.8. GraphPad Prism 9 was used for the statistical analyses of the data. All *P*-values were determined by two-tailed, unpaired Student's *t*-test with unequal variances or log rank (Mantel–Cox) test and are indicated in the figures. *P*-values of <0.05 were considered to indicate statistical significance.

## Supplementary Material

Supplement 1

Supplement 2
