# Supplementary material for: Hyd/UBR5 defines a tumor suppressor pathway that links Polycomb repressive complex to regulated protein degradation in tissue growth control and tumorigenesis
Source: Genes Dev. 2024 Jul 1;38(13-14):675–91. doi: 10.1101/gad.351856.124 (PMC11368183; doi:10.1101/gad.351856.124)
Supplement: Supplement 1 [file Supplemental_Figures.pdf]

A

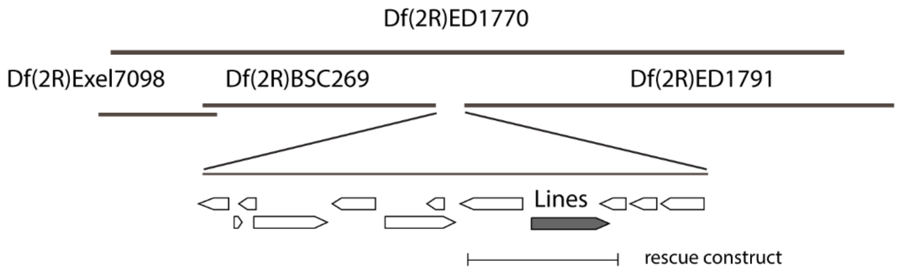

B

```
Dm   (1) MDTSSAAGSGAGTGDVCPCSTVATSTTSASNEQPQTKRQKIEQQIKVGSKALPPQPPHPPDILDLDAN
Hs   (1) ---- -----------------------------------------------------------------

Dm  (69) SNSHLCSSLSSSSSHSLSTPSTANNSPTTTPCSSRAASYAQLLHNILPQNGDTDLHSQPESAHDEVDR
Hs   (1) --------------------------------------------------------------------

Dm (137) AAKLPNLLTLDTTNGNGSHASSRSNCLQATTATSIDDMLEFEQSLTRQCLCGVSERTLRKPFQSHYSQ
Hs   (1) --------------------------------------------------------------------

Dm (205) DTNGQKRIAYLREWPTNKLLQFLSNLQLLFDIYLKQNAKGFICTRIMDVCDALIRNDHKLIDEIIVLA
Hs   (1) --------------------------------------------------------------MKVFCE

Dm (273) GYENSYVQFLAGRVLAAFLVIAKQELNDEWLQKIVDQLFNFEQLDQAAVQKIHFSLDIIKRIVEWKDM
Hs   (7) VLEELYKKVLLGATLENDSHDYIFYLNPAVSDQDCSTATSLEWANTCGIQGRHQPISVGVAPIAVAPV

Dm (341) EIHPLDDDWMASANSTSAASSVVPLSTEASVSYMHFPVQEQPLATNYFALQFREDTEGERETGQEAPD
Hs  (75) CLKTNSQMSGSREVMLLQLTVIKVMTTRILSVKTEFHAKEQYRDVIKILLESAKVDSKLICMFQNSDK

Dm (409) NRDRHRRHFGEDMNVAYEPHPAPQSTTMPSGCHVVTLTDSESFDTTHLKCITIQKLEHKWPTLVKNMS
Hs (143) LLSHMAAQCLALLLYFQLREKITLSNSWIAFCQKNLSEYSESNKAIYCLWTLTAIIKEIFKDSCSQKT

Dm (477) ELMAPTHQDAAEHCVLNFLQLWENIISVKANLSIDETRPYYAQLDKFELLLSHSLSCTVYKQMLCLFN
Hs (211) EILKQFLTHFDTIFEVFYNSLFSQHFEN-----CRDTSKIVNILMCFLDLLELLIASRIHLKLHFTCQ

Dm (545) EALCYGSTLALQDMLPEETCKLAHQIVCHVRGFRILESLPRRQPDNMVSLIGYNGKPMVYANGTITLA
Hs (274) RILFLKPSCMLEVITWPIQAFVKRKVIIFLKKCLICKVGEDLCRGSVPALMPPDHHVAVDMLALANAV

Dm (613) HAAQSGDSEEDGAPLDLIEMDKTLLQKMVLLVLKSIAVTVKEIRSDSSDSSIDSTDYDAFQDMMLIER
Hs (342) LQAVNSGLLKTLSVYEKHSFFGGDEVQPECELITSPDHVILRAASLVIMKSLEIKFQNYSSASEMKVD
                                                 *(A24)
Dm (681) SIRDVLSKLETFIKQTLEFHPECHFSKILIHLFDDQDDHLIEAMVCTLDVTSGISFRNNAFPELVAM-
Hs (410) LQRFMSELLTFLKPHLQPSLQLHNPCKWLSRVFIEQDDDMLEAAKASLGIYLTLTRGCEATESLTQGK
                                                                  *(A25)
Dm (748) -----------LNPVYTFLEFLKMTSNSSDLLLDLLVSNETCFLLYLLRLLKYIRMNWTMFVHSCHTF
Hs (478) EMWDHHTHENGYNPHCIFLFFLKNIGFDSTVLLDFLISSETCFLEYFVRYLKLLQKDWDNFFTICNNF

Dm (805) GMGSAMLDEAMGVLIRLRLQIS----------------------------------------------
Hs (546) DATESKYDISICGCVPSLVQDQSSNQTIPHRLTAPHSHRDVCARHSWASDAPSEPLKAVMSKGAHTMC

Dm (827) --------------------------------------------------------------------
Hs (614) ASSLSSPRASQSLVDYDSSDDSDVESTEQCLANSKQTSLHQQATKEIQDAAGTSRDKKEFSLEPPSRP

Dm (827) ---------------------------------------------RLVSRQLYPYDISPVLRLLESCESL
Hs (682) LVLKEFDTAFSFDCEVAPNDVVSEVGIFYRIVKCFQELQDAICRLQKKNLFPYNPTALLKLLKYIEVI

Dm (852) YEGNELS-
Hs (750) SNKTMNTL
```

Supplemental Figure 1. Genetic mapping and molecular characterization of *A24* and *A25* alleles.

(*A*) Deficiency mapping of *A24* and *A25* mutant alleles. *Df(2R)ED1770* failed to complement *A24* and *A25*, while *Df(2R)Exef7098*, *Df(2R)BSC269* and *Df(2R)ED1791* complemented *A24* and *A25*, thus placing *A24* and *A25* in a small interval between *Df(2R)BSC269* and *Df(2R)ED1791*. A schematic view of this genetic interval including *lin* and the neighboring genes is shown. Also shown is a *P[lin+]* genomic rescue transgene used in this study.

(*B*) Sequence alignment of Lin orthologue from *Drosophila melanogaster* (Dm) and *Homo sapiens* (Hs). The residues mutated in A24 and A25 are marked. The F713S mutation in A24 changed an identical residue between Dm and Hs, while the N793I mutation in A25 changes a semi-conserved residue between Dm and Hs.

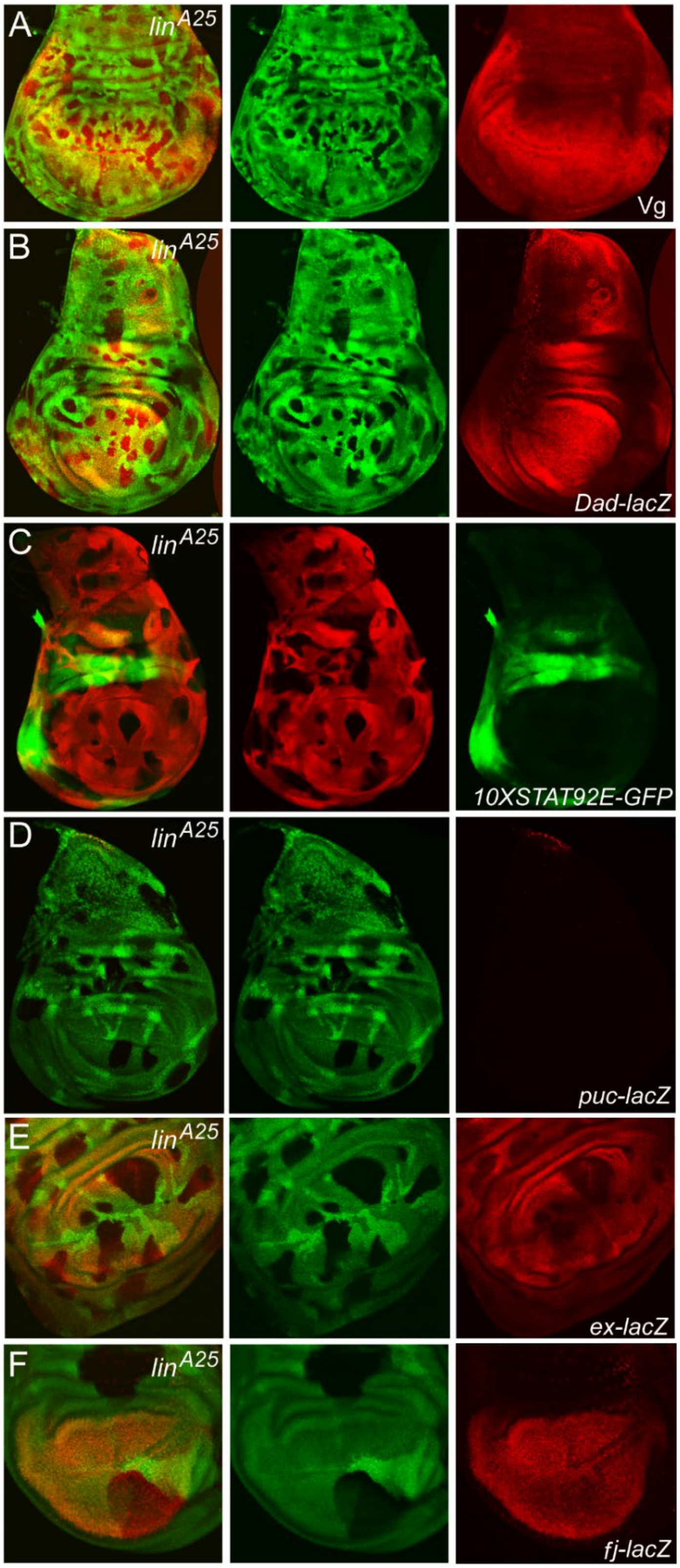

Supplemental Figure 2. Analysis of various developmental pathways in *lin*^{A25} mutant clones in wing imaginal discs.

In all panels, *lin*^{A25} mutant clones created by the FLP-FRT system were marked by absence of GFP expression (green), except in C, where *lin*^{A25} mutant clones were marked by absence of Arm-lacZ expression (red). Vg indicates Wg pathway activity, *Dad-lacZ* indicates BMP pathway activity, *10XSTST92E-GFP* indicates JAK/STAT pathway activity, *puc-lacZ* indicates JNK pathway activity. *ex-lacZ* and *fj-lacZ* indicates Hippo pathway activity.

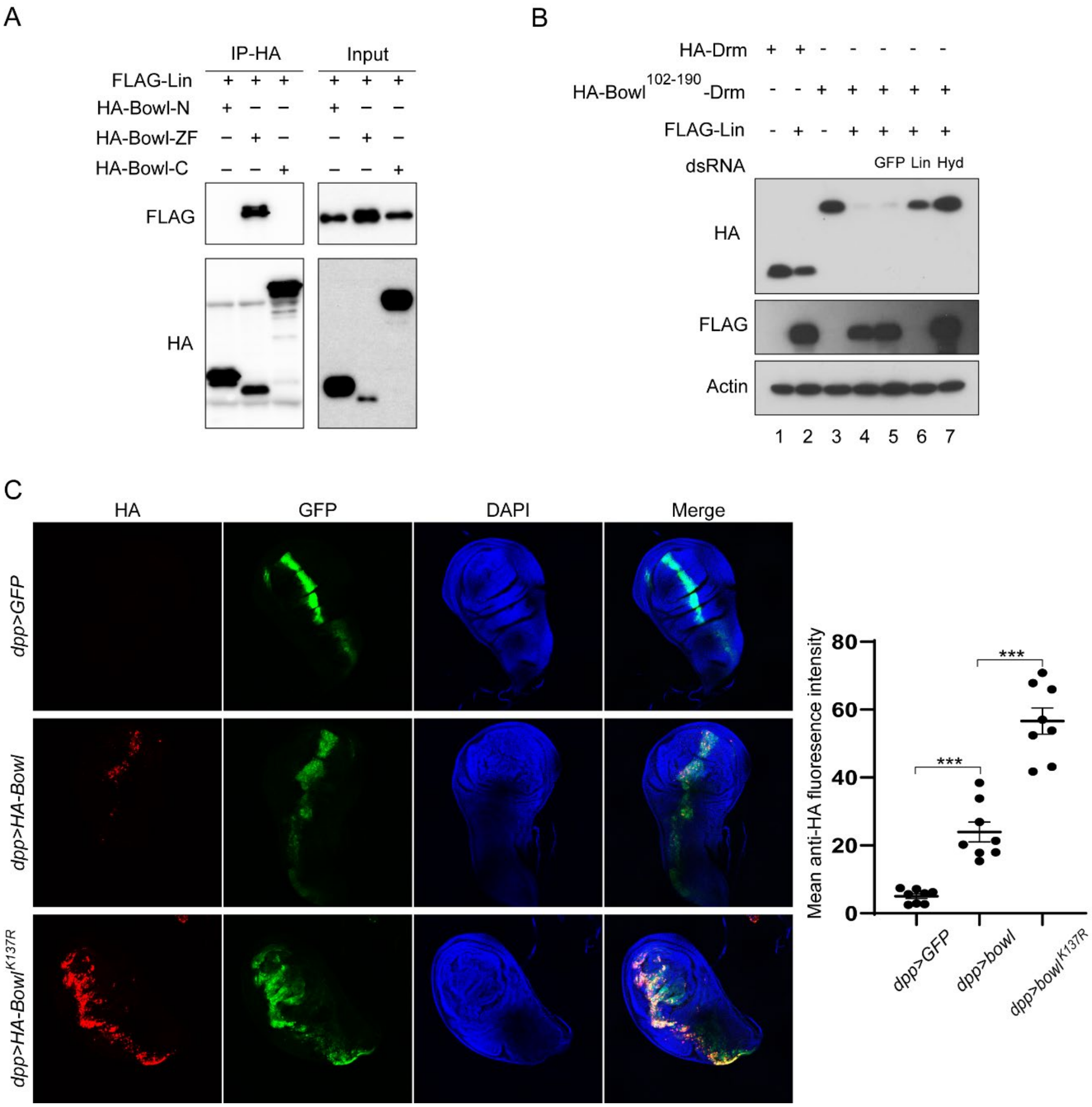

Supplemental Figure 3. Characterization of Bowl-Lin interaction and the CNM required for Bowl degradation.

(*A*) Physical interaction between Bowl ZF and Lin. S2R+ cells expressing the indicated constructs were subjected to co-IP assay as indicated. Co-IP was readily detected between Lin and Bowl ZF (lane 2), but not between Lin and Bowl N-terminus (lane 1), or between Lin and Bowl C-terminus (lane 3).

(*B*) HA-Drm or a fusion construct between the CNM of Bowl and Drm (HA-Bow1$^{102\text{-}190}$-Drm) was co-expressed with FLAG-Lin and the resulting cell lysates were probed with the indicated antibodies. Co-expression of FLAG-Lin promoted the degradation of HA-

-Bow1$^{102\text{-}190}$-Drm fusion protein, but not HA-Drm (compare lanes 1-4). Furthermore, Lin-induced degradation of HA-Bow1$^{102\text{-}190}$-Drm fusion protein was suppressed by RNAi against Hyd or Lin, but not RNAi against GFP (lanes 5-7).

(C) Third instar wing discs expressing *UAS-HA-bowl* or *UAS-HA-bowl*^{K137R} by the *dpp-Gal4 UAS-GFP* driver were stained with an anti-HA antibody and DAPI. Note the increased staining of HA-Bowl^{K137R} compared to HA-Bowl. Quantification of the mean anti-HA fluorescence intensity of *dpp* domain is shown to the right (mean ± SEM; n = 8). *** denotes a p-value<0.001.

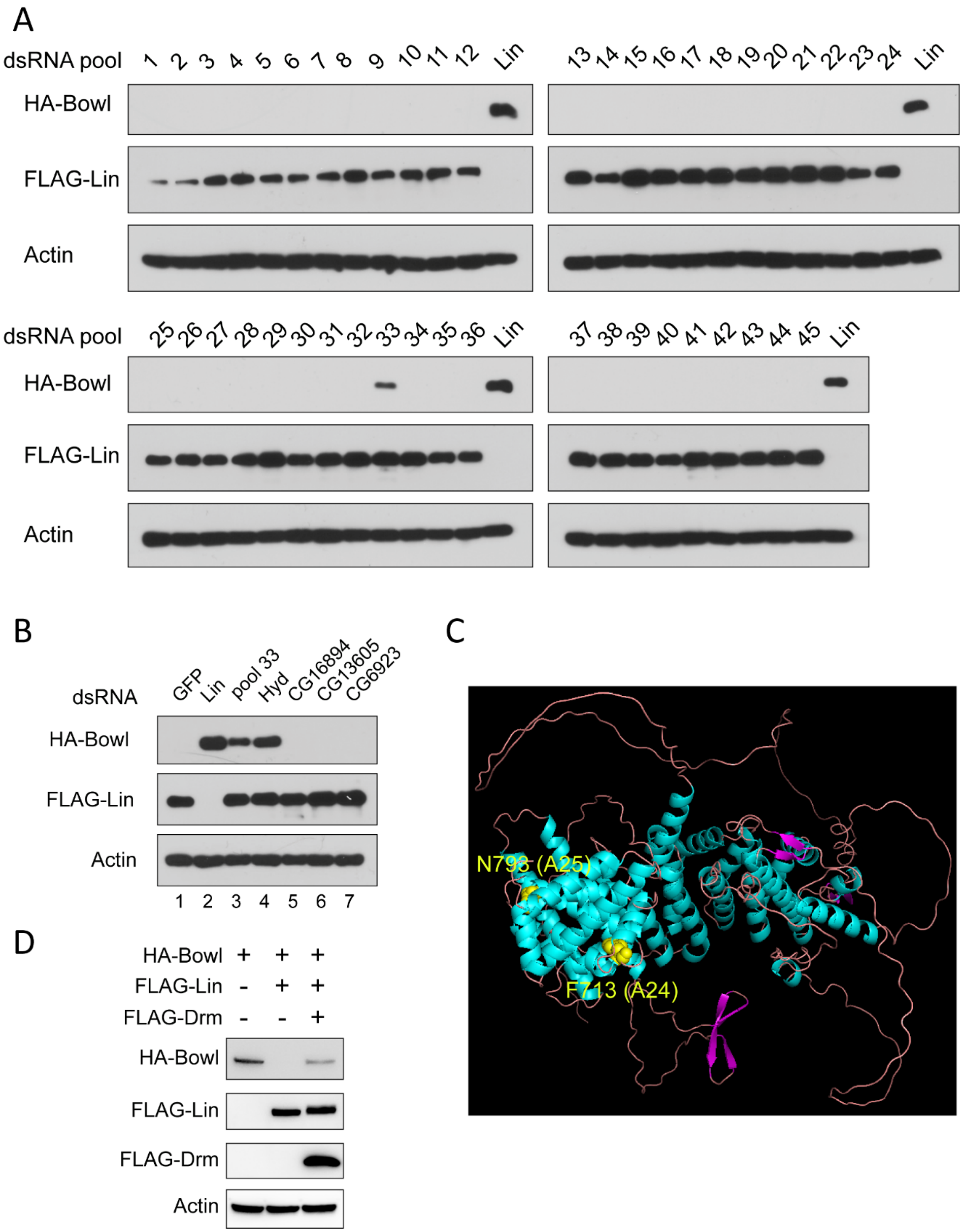

Supplemental Figure 4. A pooled RNAi screen identified Hyd as an E3 ligase required for Lin-induced Bowl degradation.

(*A*) Screening results from the pooled RNAi screen. 45 dsRNA pools, each containing dsRNAs for four different E3 ligases, were used to treat S2R+ cells expressing HA-Bowl and FLAG-Lin. HA-Bowl was normally undetectable under such condition due to Lin-induced degradation. dsRNA against Lin, which prevented Lin-mediated Bowl degradation, was included as a positive control in the screening (rightmost lane in each gel). Pool #33 was the only dsRNA pool that stabilized HA-Bowl. Also note that pool #33 did not affect FLAG-Lin protein level.

(*B*) Identification of Hyd as the responsible E3 ligase within pool #33 that is required for Lin-induced Bowl degradation. Individual dsRNA within pool #33 (Hyd, CG16894, CG13605, or CG6923) was used to treat S2R+ cells expressing HA-Bowl and FLAG-Lin. HA-Bowl was normally undetectable under such conditions due to Lin-induced degradation. Only Hyd dsRNA stabilized HA-Bowl. Also included are positive control (Lin dsRNA) and negative control (GFP dsRNA).

(*C*) 3D structure model of Lin predicted by AlphaFold. The two critical residues F713 (Lin^{A24}) and N793 (Lin^{A25}) identified in the forward genetic screening are highlighted in yellow color.

(*D*) Drm inhibited Lin-induced Bowl degradation. S2R+ cells expressing the indicated constructs were analyzed for Bowl expression. Note that Lin-induced Bowl degradation was largely suppressed by Drm expression.

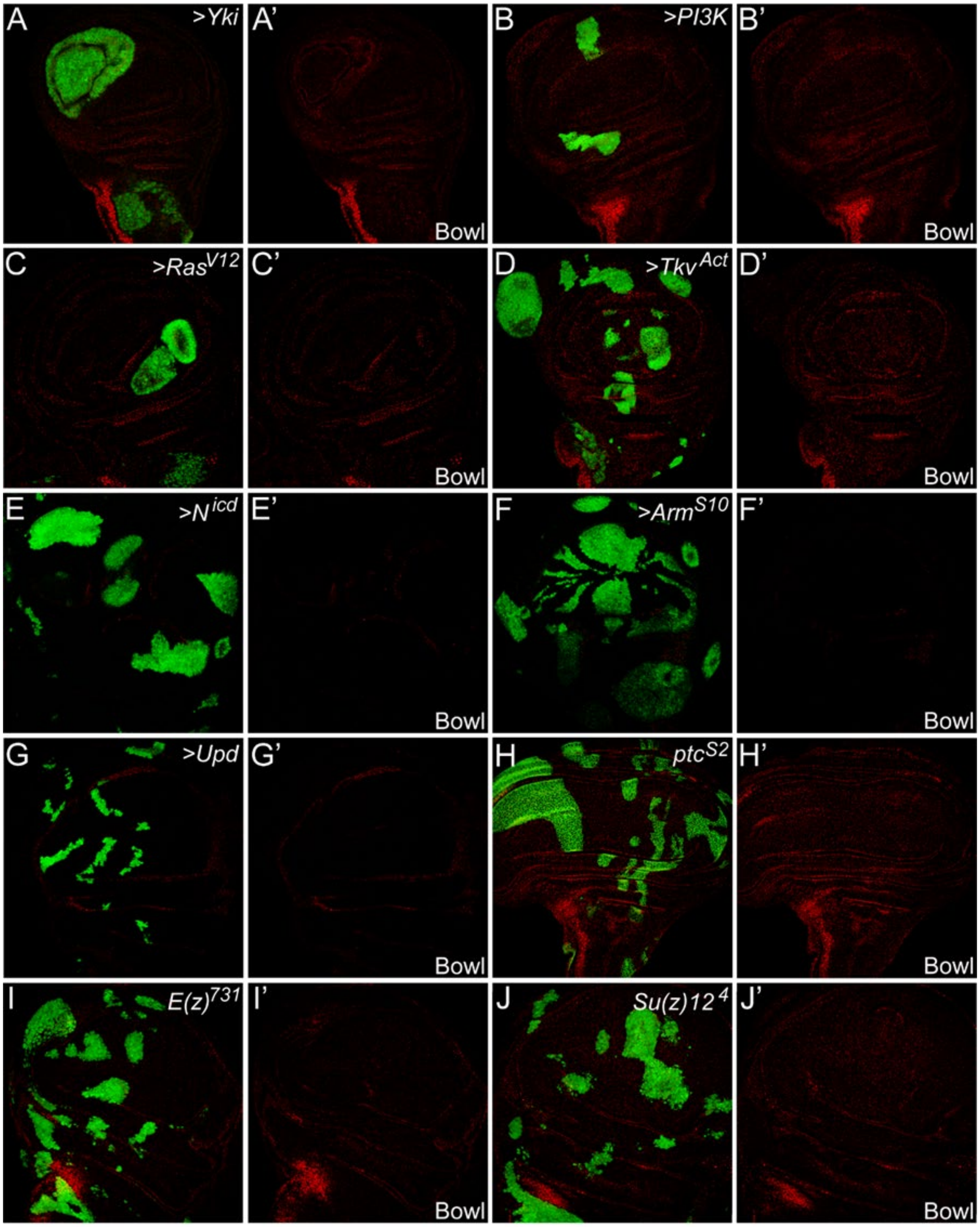

Supplemental Figure 5. The effect of known signaling pathways on Bowl protein accumulation.

Third instar wing discs containing GFP-positive MARCM clones of the indicated genotype were stained for Bowl protein (red). No accumulation of Bowl was observed in any of the mutant clones.

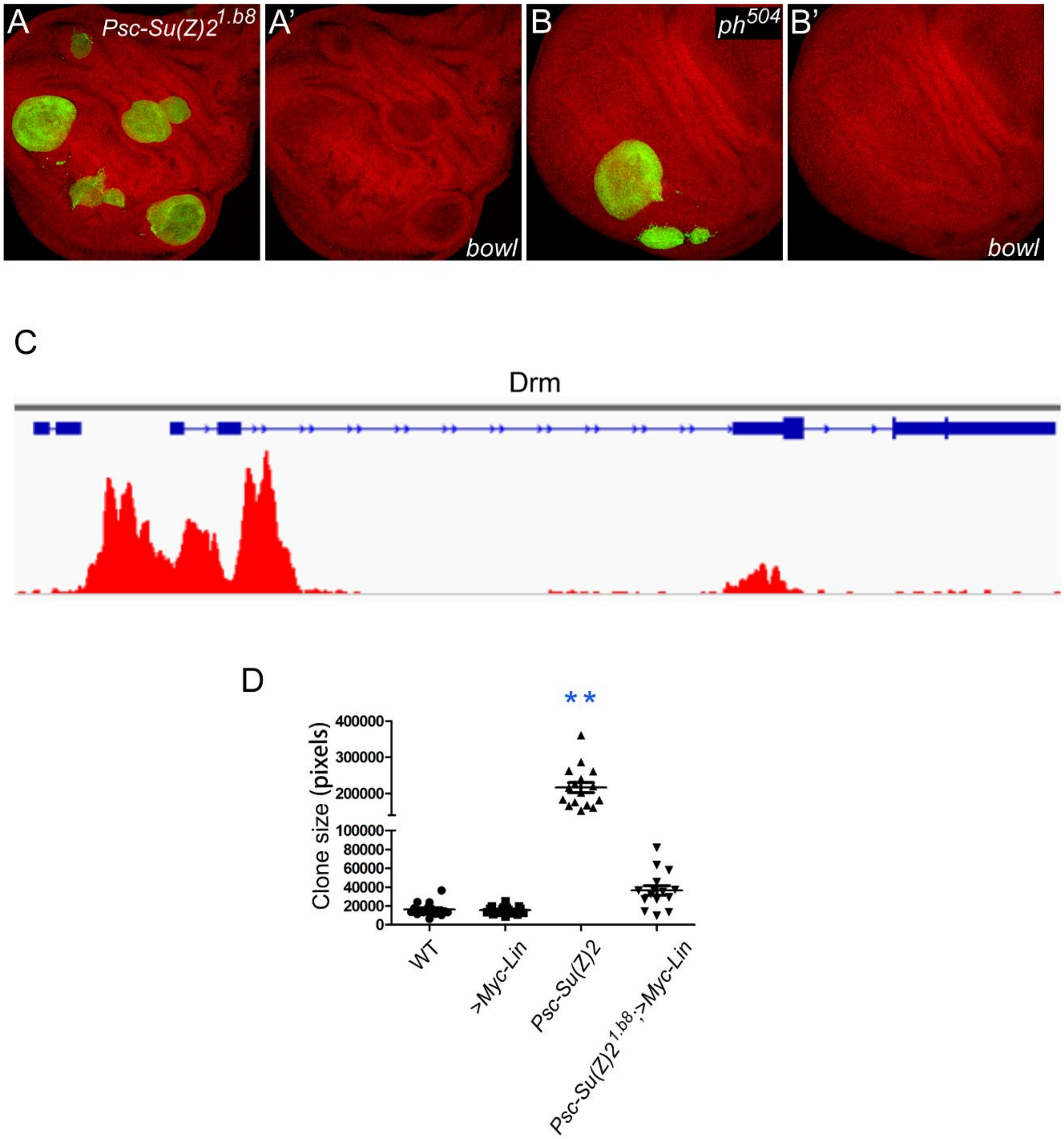

Supplemental Figure 6. Analysis of Bowl mRNA levels in PRC1 mutant clones and quantification of clone size from Fig. 6E-H.

(*A-B'*) Third instar wing disc containing GFP-positive MARCM clones of *Psc-Su(Z)2*$^{1.b8}$ (*A-A'*) or *ph*^{504} (*B-B'*) was analyzed for *bowl* mRNA expression by FISH (red). Note that *bowl* mRNA level was not increased in both mutant clones compared to the neighboring wildtype cells.

(*C*) Distribution of the PRC1 component Polyhomeotic (PH) at the genomic locus of *drm* calculated from genome-wide ChIP-Seq data of *Drosophila* embryos (Schuettengruber et

al. 2014). Arrows point to the direction of transcription. Note that PH binding is highly enriched at promoter and 5’ UTR.

(*D*) Quantification of clone size in experiments described in Fig. 4E-H. The data are mean ± SEM; $n \geq 15$. ** denotes a p-value<0.01.

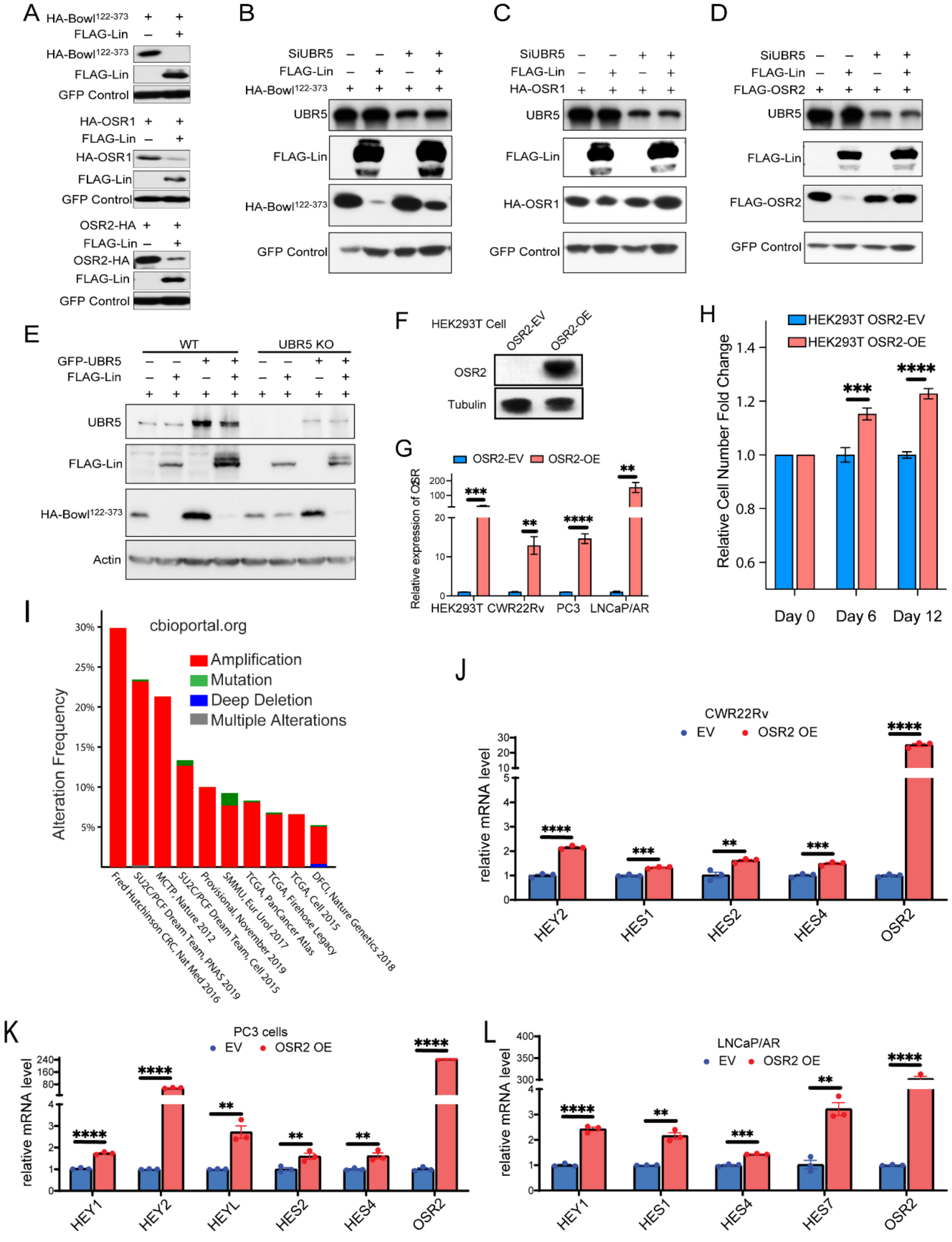

Supplemental Figure 7. Functional conservation of UBR5 and OSR1/2.

(*A*) HEK293 cell lysates expressing the indicated constructs were analyzed by western blotting. Note the dramatic reduction of HA-Bowl122-373, HA-OSR1 and OSR2-HA

when FLAG-Lin was co-expressed. A co-transfected GFP construct was used as a control.
(*B*) HEK293 cell lysates expressing the indicated constructs were analyzed by western blotting. Note FLAG-Lin-induced HA-Bowl122-373 reduction was suppressed by UBR5 RNAi.
(*C*) HEK293 cell lysates expressing the indicated constructs were analyzed by western blotting. Note FLAG-Lin-induced HA-OSR1 reduction was suppressed by UBR5 RNAi.
(*D*) HEK293 cell lysates expressing the indicated constructs were analyzed by western blotting. Note FLAG-Lin-induced FLAG-OSR2 reduction was suppressed by UBR5 RNAi.
(*E*) HEK293 cell lysates expressing the indicated constructs were analyzed by western blotting. Note the dramatic reduction of HA-Bowl122-373 when co-expressed with FLAG-Lin in wildtype, but not UBR5 knockout cells. Also note that Lin-induced decrease of HA-Bowl122-373 was restored by re-introduction of GFP-UBR5 in UBR5 knockout cells.
(*F*) Western blot of OSR2 and Tubulin control in HEK293T cells transduced with the indicated constructs.
(*G*) Relative expression level of OSR2 in various human cell lines transfected with the indicated constructs. mean ± SEM, n=3; p values were calculated using multiple t-test.
(*H*) Relative cell number fold change of HEK293T cells transfected with the indicated constructs, normalized to OSR2-EV (empty vector) group, measured in a FACS-based competition assay. OSR2-OE denotes OSR2 overexpression. mean ± SEM, n=3; p values were calculated using multiple t-test.
(*I*) Frequency of OSR2 genomic alterations in different prostate cancer genomic studies, figure adapted from cbioportal.org.
(*J-L*) OSR2 overexpression upregulated Notch pathway target genes 63,64 in prostate cancer cell lines CWR22Rv, PC3 and LNCaP/AR. mean ± SEM, n=3; p values were calculated using multiple t-test.
For all panels, mean ± s.e.m. is represented and **** represents p<0.0001. *** p<0.001. ** p<0.01. * p<0.05.
